# Regulation of epitope exposure in the gp41 membrane-proximal external region through interactions at the apex of HIV-1 Env

**DOI:** 10.1371/journal.ppat.1010531

**Published:** 2022-05-18

**Authors:** Hannah M. Schapiro, Mukta D. Khasnis, Koree Ahn, Alexandra Karagiaridi, Stephanie Hayden, Maria E. Cilento, Michael J. Root

**Affiliations:** 1 Department of Biochemistry and Molecular Biology, Sidney Kimmel Medical College, Thomas Jefferson University, Philadelphia, Pennsylvania, United States of America; 2 Department of Microbial Infection and Immunity, The Ohio State University College of Medicine, Columbus, Ohio, United States of America; Loyola University Chicago, UNITED STATES

## Abstract

Glycoprotein Env of human immunodeficiency virus type 1 (HIV-1) mediates viral entry through membrane fusion. Composed of gp120 and gp41 subunits arranged as a trimer-of-heterodimers, Env adopts a metastable, highly dynamic conformation on the virion surface. This structural plasticity limits the temporospatial exposure of many highly conserved, neutralizing epitopes, contributing to the difficulty in developing effective HIV-1 vaccines. Here, we employed antibody neutralization of HIV-1 infectivity to investigate how inter- and intra-gp120 interactions mediated by variable loops V1/V2 and V3 at the Env apex regulate accessibility of the gp41 membrane-proximal external region (MPER) at the Env base. Swapping the V3 loop from Env_SF162_ into the Env_HXB2_ background shifted MPER exposure from the prefusogenic state to a functional intermediate conformation that was distinct from the prehairpin-intermediate state sensitive to gp41-targeted fusion inhibitors. The V3-loop swap had a profound impact on global protein dynamics, biasing the equilibrium to a closed conformation resistant to most anti-gp120 antibodies, stabilizing the protein to both cold- and soluble CD4-induced Env inactivation, and increasing the CD4 requirements for viral entry. Further dissection of the Env_HXB2_ V3 loop revealed that residue 306 uniquely modulated epitope exposure and trimer stability. The R306S substitution substantially decreased sensitivity to antibodies targeting the gp41 MPER and, surprisingly, the gp120 V3-loop crown (residues 312–315), but had only modest effects on exposure of intervening gp120 epitopes. Furthermore, the point mutation reduced soluble CD4-induced inactivation, but had no impact on cold inactivation. The residue appeared to exert its effects by electrostatically modifying the strength of intra-subunit interactions between the V1/V2 and V3 loops. The distinct patterns of neutralization and stability pointed to a novel prefusogenic Env conformation along the receptor activation pathway and suggested that apical Env-regulation of gp41 MPER exposure can be decoupled from much of the dynamics of gp120 subunits.

## Author summary

Surface glycoprotein Env is the main target for neutralizing antibodies elicited by HIV-1 vaccines. Env spontaneously fluctuates among different structures, limiting exposure of many attractive antibody-binding epitopes and, thereby, confounding vaccine development. To characterize these fluctuations, we examined how exposure of the MPER epitope found at the base of Env is regulated by interactions of the V3 loop located in the apex. Starting with an extremely flexible Env with a readily-exposed MPER, we identified two alterations that substantially restricted antibody access to the epitope. The first, a wholesale swap of V3 loops between HIV-1 strains, energetically stabilized Env in a closed structure that restricted access to antibodies throughout the protein. The second, a point mutation that altered V3-loop charge, specifically destabilized the MPER-exposed conformation but had minimal impact on antibody access to Env regions in between the apex and base. The results indicate that MPER exposure is not explicitly tied to the dynamics of Env regions between the apex and base and suggest a new structural fluctuation during Env activation.

## Introduction

The Envelope glycoprotein (Env) from human immunodeficiency virus type 1 (HIV-1) is responsible for mediating viral entry through membrane fusion [1]. As the sole viral protein on the virion surface, Env is the primary focus of vaccine development efforts to generate neutralizing antibodies (NAbs) that block viral replication [2]. Unfortunately, these efforts have proven challenging for a number of reasons, including the dense glycan shield on Env providing a flexible barrier to the underlying protein, the breadth of Env sequence diversity in the general population, the speed at which resistant Env species emerge within an individual, and the conformational plasticity of Env that temporally limits exposure of conserved epitopes critical for function [3–5]. In this study, we further investigated the regulation of this conformational flexibility by exploring how inter- and intrasubunit interactions at the apex of Env control NAb epitope exposure at the base of the complex.

Env is a homotrimeric, single-pass transmembrane protein with each protomer consisting of two subunits: surface subunit gp120 responsible for binding cellular CD4 and chemokine receptors (CCR5 and/or CXCR4, collectively denoted CoR), and transmembrane subunit gp41 responsible for catalyzing membrane fusion [6]. In the native state, the gp120 subunits form a canopy that covers much the gp41 trimer, with extensions of each gp120 subunit tightly enmeshed with its own gp41 elements at the base (juxtamembrane portion) of the complex [7–9]. The trimeric organization is stabilized by gp120-gp120 interactions involving variable loops V1/V2 and V3 at the apex and by a coiled coil formed by part of the gp41 N-terminal heptad repeat (N-HR) at the base.

In the presence of target cell membranes, CD4 binding to the gp120 canopy disrupts V1/V2- and V3-loop interactions, leading them to disengage from the conserved Env core as the top of each gp120 subunit rotates and relaxes radially outward [10–14]. This general opening of the Env structure releases steric constraints on several neutralizing epitopes and exposes a number of underlying conserved features of functional importance, including the gp120 bridging sheet [15–17]. Interactions of the bridging sheet and V3 loop with CoR trigger irreversible conformational changes in the gp41 trimer that uncouple it from the gp120 subunits [18–22]. First, gp41 extends into its prehairpin-intermediate (PHI) conformation with its N-terminus inserted into the target cell membrane [23–25]. Then, owing to the strong attraction of the N-HR coiled coil and C-terminal heptad repeat regions (C-HR), gp41 collapses into its trimer-of-hairpins (TOH) structure that positions viral and cellular membranes for fusion [26–30].

**Fig 1:**
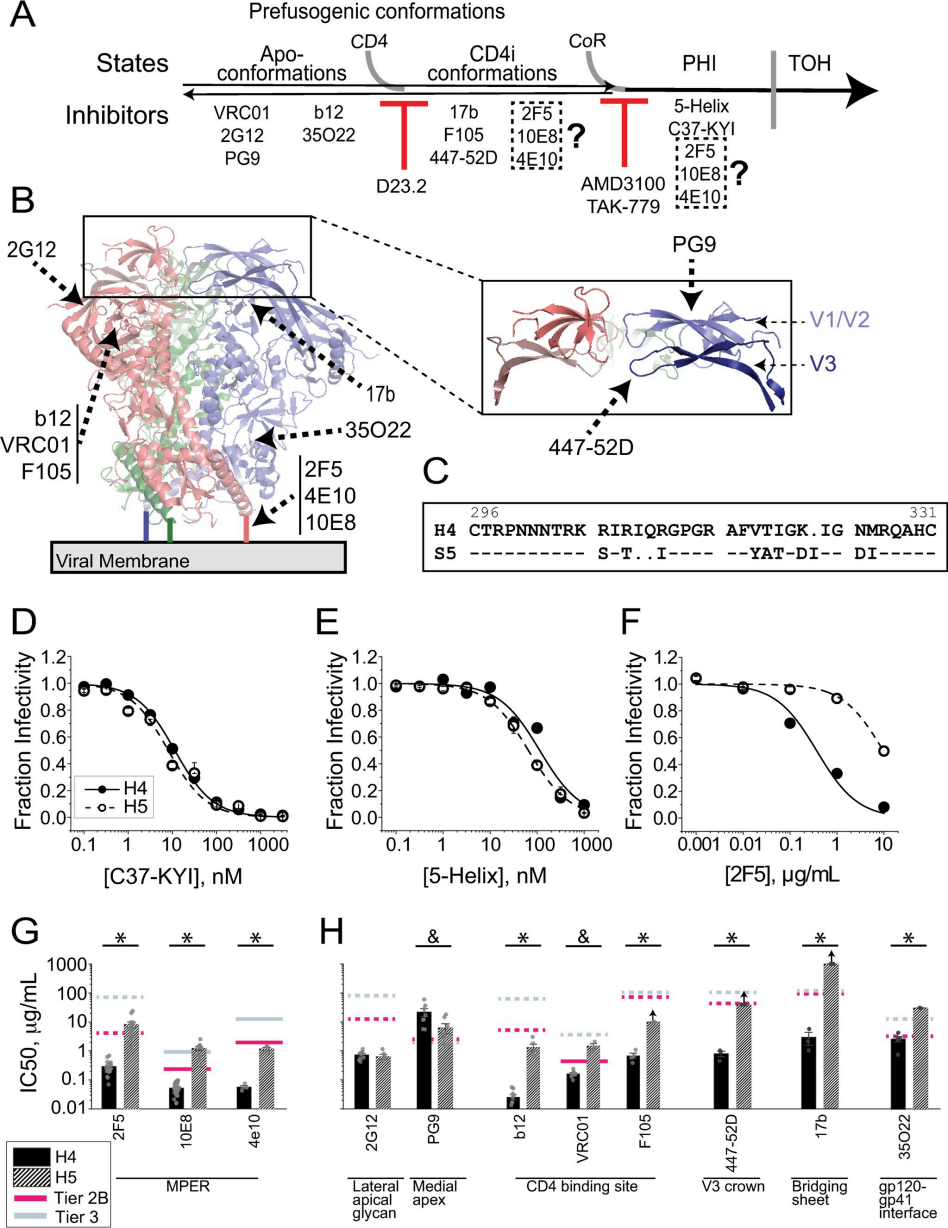
Swapping the Env_SF162_ V3 loop into Env_HXB2_ significantly alters glycoprotein conformational equilibrium. (**A**) Timeline of Env structural transitions leading to membrane fusion. The prefusogenic state reversibly fluctuates through a number of apo- and CD4-induced (CD4i) conformations. CD4 binding preferentially stabilizes the CD4i conformations, while chemokine-receptor (CoR) binding triggers transition into the prehairpin intermediate state (PHI). The PHI ultimately collapses into the fusogenic trimer-of-hairpins (TOH) structure. The placement of entry inhibitors and NAbs along the timeline (bottom) indicates the approximate point at which each one blocks downstream conformational transitions. (**B**) Ribbon diagram of the Env_JRFL_ SOSIP.664 structure (PDB 5FYK [79]) with each protomer illustrated in a different color. The V1/V2 and V3 loops are shown in isolation (enlargement). The approximate locations of neutralizing epitopes are indicated for the NAbs used in this study. (**C**) Sequence alignment of the V3 loops from Env_HXB2_ and Env_SF162_ (numbering according to the Env_HXB2_ sequence). Dash (“-“) represents identically conserved amino acids, while dot (“.”) indicates a sequence gap. (**D-F**) Inhibitory titrations of fusion inhibitors C37-KYI (**D**) and 5-Helix (**E**) and anti-gp41 MPER bNAb 2F5 (**F**) against HIV-1 pseudotyped with either wild type Env_HXB2_ (H4, filled circles)) or V3-loop chimeric H5 Env (open circles). (**G-H**) Sensitivity of H4 (solid) and H5 (hatched) Envs to antibodies targeting the MPER (**G**) or other regions of the glycoprotein trimer (**H**). The average IC50 values for each NAb are compared to the geometric means of IC50 values against tier 2-clade B (magenta lines) and tier 3 (slate lines) Envs (compiled from LANL HIV Immunology Database; lines are solid or dashed depending, respectively, on whether >85% or <85% of isolates were reportedly neutralized). Arrows above the bars for antibodies F105, 447-52D and 17b against H5 Env indicate that 50% neutralization was not achieved at the highest concentrations tested. Data points in **D-F** and bars in **G-H** represent the mean±SEM from three or more independent experiments. Single asterisk in **G-H** indicates a significant difference as determined by a two-sample t-test (equal variance not assumed) with p < 0.05; ampersand (for NAbs PG9 and VRC01) denotes a p-value between 0.050 and 0.051.

Refolding into the TOH supplies the energy required for membrane fusion [31]. Accordingly, prefusogenic Env is metastable and, therefore, prone to dynamic fluctuation [7,32–34]. At least three distinct conformational states have been identified by single-molecule spectroscopy of unliganded Env trimers in virions [35–37]. These spontaneous fluctuations seem to emulate reversible receptor-induced conformational changes that coordinate viral entry [38]. The degree of dynamism is reflected in Env sensitivity to anti-HIV-1 NAb panels (Fig 1A and 1B) [2,35,39,40]. Primary isolate Envs that are broadly resistant to antibody neutralization tend to explore less conformational space than their structurally-labile, antibody-sensitive, laboratory-adapted counterparts [41,42]. Spontaneous fluctuations into epitope-accessible open conformations are more energetically unfavorable for primary isolate Envs, with their decreased sensitivity to temperature- and soluble CD4 (sCD4)-induced Env inactivation and increased requirement of cellular CD4 for viral entry attributed to this energy barrier [22,35,36,43–47]. Predictably, mutations that disrupt closed conformations have an overall destabilizing effect on the complex, resulting in enhanced conformational flexibility and increased NAb sensitivity [43,48–57].

Here, we used NAb sensitivity profiles and stability readouts to interpret how alterations at the Env apex impacted the prefusogenic conformational equilibrium, focusing especially on exposure of the gp41 membrane-proximal external region (MPER) at the trimer base. Previous work has shown that mutations in the V1/V2 and V3 loops of primary isolate Envs can destabilize the closed conformation, increasing temporospatial exposure of neutralizing epitopes throughout the trimer, including the MPER [48,53,57–62]. In this study, we sought to accomplish the opposite. We started with NAb-sensitive, laboratory-adapted isolate Env_HXB2_ and constrained its conformational dynamics through two different modifications to its V3 loop: swapping the entire V3 loop with that from a different Env strain or introducing a charge-altering mutation at V3-loop residue 306. Both modifications substantially reduced anti-MPER antibody potency by shifting MPER exposure from the prefusogenic conformation to a CD4-induced intermediate state. The V3-loop swap produced a chimeric Env variant with the broad neutralization resistance, stability profile and CD4 utilization characteristic of primary isolate Envs. By contrast, the point mutation conferred resistance to sCD4 inactivation and increased the requirement of cellular CD4 for viral entry, but it minimally perturbed exposure of many gp120 epitopes and had no impact on temperature-induced inactivation. The results imply that exposure of the Env gp41 base can be regulated independently from much of the dynamics of the gp120 canopy, motivating us to postulate an additional state along the Env activation pathway leading to membrane fusion.

## Results

### A V3-loop swap in Env_HXB2_ globally impacts trimer structure without altering late conformational changes that drive membrane fusion

We previously engineered the chimeric H5 Env by swapping the V3 loop from CCR5-tropic Env_SF162_ (hereafter denoted S5 Env) into the CXCR4-tropic Env_HXB2_ (denoted H4 Env) [63,64]. The V3 loops from H4 Env and S5 Env are comprised of 36 and 35 amino acids, respectively, and differ at 10 residue positions (Fig 1C). The largest physiochemical change is a net charge reduction from +9 for HXB2 to +3 for SF162. Unlike the parental H4 Env, chimeric H5 Env was poorly fusogenic on CD4^+^CXCR4^+^CCR5^-^ cells but mediated robust fusion on CCR5-expressing target cells (S1 Fig) [64].

Despite switching CoR tropism, the V3-loop swap did not exert a dramatic effect on late Env conformational changes triggered by CoR binding. H4 and H5 Envs were similarly sensitive to fusion inhibitors (FIs) C37-KYI and 5-Helix, implying that the spatiotemporal exposure of the gp41 N-HR and C-HR regions during the PHI were not substantially altered (Fig 1D and 1E) [65,66]. Curiously, inserting the SF162 V3 loop into Env_HXB2_ led to a 29-fold reduction in the potency of NAb 2F5, whose extended epitope at the junction of the gp41 C-HR and MPER regions partially overlaps the binding site for 5-Helix (Fig 1F) [67]. Comparable reductions in potency were observed for NAbs 10E8 and 4E10, which target MPER segments C-terminal to that for NAb 2F5 (Fig 1G). In fact, the IC50 values measured for these anti-MPER NAbs against H5 Env were close to or exceeded the geometric mean IC50 value reported for tier 2 clade B isolates [S1 Table, data from the Los Alamos National Laboratory (LANL) HIV Immunology Database, January 2022]. The loss of potency did not appear to reflect reduced binding strength, as neutralization was effectively irreversible for HIV-1 pseudotyped with either H4 or H5 Env in antibody dilution assays (S2 Fig). Rather, our results suggested H4 and H5 Envs substantially differ in temporospatial exposure of the MPER, mirroring previous observations that showed mutations at the Env apex can exert dramatic effects on the juxtamembrane region over 90 Å away [48].

To assess the global impact of the V3-loop swap on Env structure, we compared sensitivities of H4 and H5 Envs to a panel of neutralizing antibodies with epitopes throughout the glycoprotein (Fig 1B and S1 Table). H5 Env was significantly less sensitive to antibodies targeting most tertiary and quaternary epitopes, including the V3 crown (the -GPGR- sequence invariant to the V3-loop swap), the CD4 binding site, the bridging sheet exposed in the CD4-induced (CD4i) conformation, and the gp120-gp41 interface (Fig 1H). Notably, the potency of anti-CD4i antibody 17b was reduced more than 300-fold by the V3-loop swap. Two exceptions to this trend were NAbs 2G12 and PG9. NAb 2G12 targets lateral surface glycans at residue positions unaffected by the V3-loop swap [68,69]; not surprisingly, the antibody neutralized H4 and H5 Envs similarly. NAb PG9 targets a quaternary epitope of V1/V2 loops most stabilized in apo-Env conformations [2,70,71]; interestingly, this antibody had more potent activity against H5 Env than H4 Env. Overall, the neutralization profile of H5 Env resembled that for tier 2 clade B primary isolate Envs with closed, epitope-occluded prefusogenic conformations [39]. Such structures are substantially different from the open, easily neutralizable conformations of tier 1 laboratory-adapted Envs like H4.

### The V3-loop swap modifies temporal properties of MPER exposure

**Fig 2:**
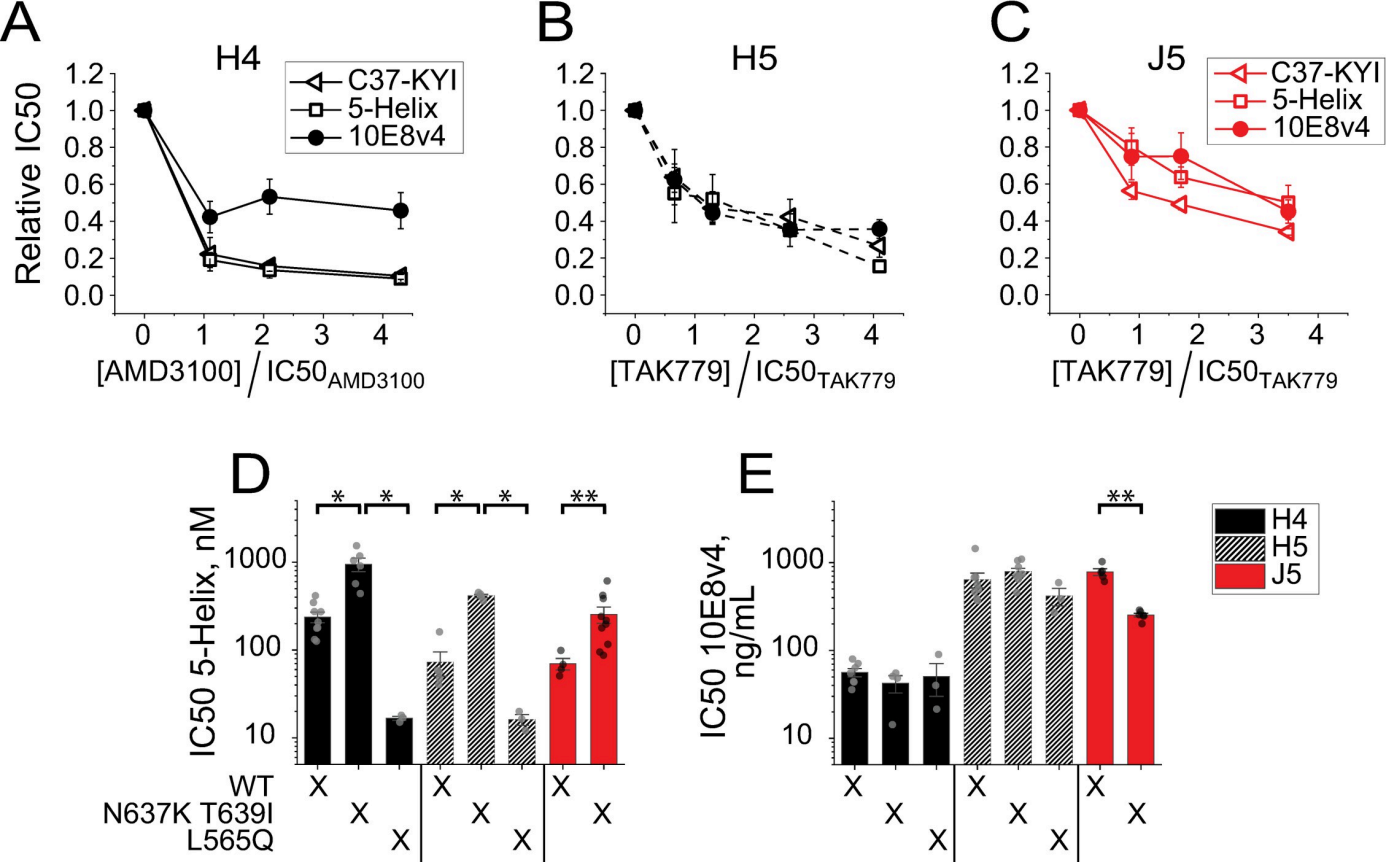
The V3-loop swap alters the temporospatial exposure of the MPER epitope in Env_HXB2_. (**A-C**) Impact of CoR antagonism on the potencies of gp41-targeted inhibitors against HIV-1 pseudotyped with H4 Env (**A**), H5 Env (**B**) or J5 Env (**C**). IC50 values for C37-KYI (open triangles), 5-Helix (open squares) and NAb 10E8v4 (filled circles) were determined in the presence of either AMD3100 (**A**) or TAK-779 (**B, C**). Data have been normalized to the IC50 levels obtained in the absence of CoRA. Note that CoRA concentration is expressed relative to the CoRA IC50 for each virus (94, 61 and 23 nM for H4, H5 and J5 Envs, respectively). Data points represent the mean±SEM of four independent experiments. For H4 Env, the NAb 10E8v4 data set is significantly different (p < 0.025) from the 5-Helix data set as determined by a two-way ANOVA; for the comparison of 10E8v4 and C37-KYI data sets, p = 0.029. No statistical difference was observed in any of the data set comparisons for H5 and J5 Envs. (**D, E**) Potencies of fusion inhibitor 5-Helix (**D**) and NAb 10E8v4 (**E**) against Envs with altered PHI lifetimes. IC50 values were determined against HIV-1 pseudotyped with H4 Env (solid black), H5 Env (hatched black) and J5 Env (red) containing either the rate-enhancing N637K/T639I substitution or the rate-retarding L565Q substitution. Bars represent the mean±SEM of three or more independent experiments. Single asterisks indicate significance as determined by one-way ANOVA with means comparisons using the Tukey method with p < 0.025 [109]; double asterisks indicate significance as determined by a two-sample t-test (equal variance not assumed) with p < 0.01.

The exact timing of anti-MPER antibody binding to Env when neutralizing HIV-1 infectivity remains unclear. Some studies demonstrate a correlation between neutralization potency and MPER accessibility in the absence of target cells, suggesting the interaction occurs with the prefusogenic state [17,22,48]. Other studies show that MPER exposure is transiently enhanced following receptor activation of Env, suggesting that the interaction occurs in a fusion-intermediate state like the PHI [72,73]. To test these models, we determined how NAb 10E8 potency depended on fusion rate, as modified by CoR antagonists (CoRAs). CoRAs reduce CoR binding stoichiometry with the Env trimer, thereby prolonging intermediate state lifetimes [64,74]. As shown previously, CoRAs enhanced the potency of bona fide intermediate-state FIs C37-KYI and 5-Helix in a dose-dependent manner (Fig 2A–2C). For NAb 10E8, however, the degree of CoRA-dependent enhancement varied depending on Env strain. Against H4 Env, the enhancement of 10E8 potency was significantly lower than that for FIs (Fig 2A). By contrast, against H5 Env and J5 Env (Env_JRFL_, a representative tier 2 clade B primary isolate strain), the enhancement of 10E8 potency matched that for FIs (Fig 2B and 2C). The data suggested that much of NAb 10E8 binding to H4 Env occurs in the prefusogenic state outside the context of viral membrane fusion, while the majority of 10E8 neutralization of H5 and J5 Envs occurs during a functional intermediate state of viral entry with a lifetime dependent on CoR binding. This shared CoRA-dependence to NAb 10E8 neutralization for both H5 and J5 Envs underscored the tier 2-like characteristics conferred by the V3-loop swap in the Env_HXB2_ background.

To test whether NAb 10E8 and FIs interact with gp41 during the same intermediate state, we introduced mutations that intrinsically altered the lifetime of the PHI. The double substitution N637K/T639I in the gp41 C-HR segment shortened PHI lifetimes [64], leading to a 3- to 6-fold increase in the 5-Helix IC50 values for H4, H5, and J5 Envs (Fig 2D). Conversely, the single substitution L565Q in the gp41 N-HR segment lengthened the PHI lifetime [64], leading to a 13-fold and 4.4-fold decrease in 5-Helix IC50 values for H4 and H5 Envs, respectively. Neither substitution significantly altered NAb 10E8 potency against H4 Env (Fig 2E), as we had expected since the MPER epitope appears to be accessible for antibody binding outside the context of membrane fusion. Curiously, the mutations also had minimal impact on NAb 10E8 potency against H5 Env; moreover, the rate enhancing N637K/T639I substitution actually increased J5 Env sensitivity to NAb 10E8 neutralization. These observations implied that either the functional intermediate state targeted by NAb 10E8 does not temporally overlap with the PHI or the PHI duration constitutes only a small fraction of the NAb 10E8-neutralization window.

### The V3-loop swap significantly stabilizes the apo-conformation of the Env_HXB2_ trimer

**Fig 3:**
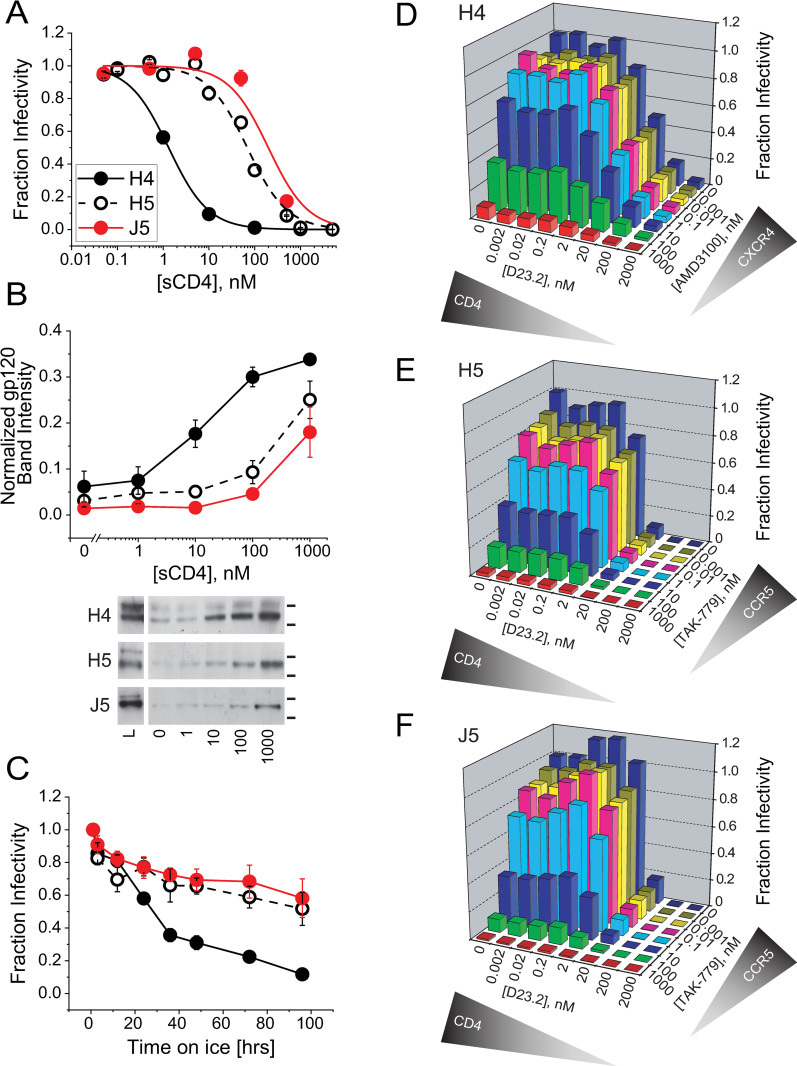
Chimeric H5 Env exhibits stability and CD4 sensitivity on par with tier 2-clade B primary isolate Env_JRFL_ (J5). (**A**) Inhibitory titrations of soluble CD4 (sCD4) against HIV-1 pseudotyped with H4 Env (black filled symbols), H5 Env (black open symbols) or J5 Env (red symbols). (**B**) Soluble CD4 dependence to gp120 shedding from H4, H5 and J5 Env-expressing HEK293T cells. Shed gp120 was detected by SDS-PAGE/Western blot, quantified by densitometry and normalized to the total gp120 detected in cell lysates. In the images of representative Western blots (bottom), L denotes a 1:3 dilution of cell lysate; the lines to the right of the images indicate positions of 160 and 100 kDa molecular weight markers. (**C**) Time dependence to cold inactivation of HIV-1 pseudotyped with H4, H5 or J5 Env. Data for each virus were normalized to the infectivity detected after a one-hour incubation on ice. (**D-F**) Dependence of viral infectivity on CD4 and CoR levels for HIV-1 pseudotyped with H4 Env (**D**), H5 Env (**E**) and J5 Env (**F**). Target cells were U87.CD4.CXCR4 (**D**) and U87.CD4.CCR5 (**E, F**). Receptors levels were titrated by adding CD4 antagonist D23.2 and CoRA AMD3100 (**D**) or TAK-779 (**E,F**) alone or in combination during the infection. Data have been normalized to the infectivity level in the absence of either receptor antagonist. Data points in **A-C** and bars in **D-F** represent the mean±SEM from three or more independent experiments (error bars for **D-F** can be found in S4 Fig). In **B** and **C**, data sets for H4 Env are significantly different (p < 0.01) from data sets for H5 and J5 Env as determined by two-way ANOVA. The H5 and J5 data sets were not statistically different.

To assess how the V3-loop swap impacted the prefusogenic Env_HXB2_ conformational equilibrium, we first explored Env sensitivity to sCD4. As an inhibitor of viral infectivity, sCD4 had more than 50-fold lower potency against H5 Env compared to H4 Env (Fig 3A), on par with the reduction observed in the potencies of NAbs targeting the CD4 binding site (Fig 1H). Likewise, the sCD4 dependence to gp120 shedding was shifted to greater than 10-fold higher concentrations for H5 Env (Fig 3B). These changes were quantitatively similar to the differences between H4 and J5 Env and, together, implied that the V3-loop swap made fluctuations into CD4-bound conformations significantly more unfavorable.

Decreased sensitivity to sCD4-induced gp120 shedding is historically associated with increased Env thermal stability [43,55,75]. To test for this correlation, we examined how the V3-loop swap altered heat and cold inactivation of viral entry. While heat inactivation did not distinguish between H4, H5 and J5 Envs (S3 Fig), differences were readily apparent in cold inactivation experiments. H5 and J5 Envs closely tracked with one another, maintaining greater than 50% infectivity after 96 hours at 0°C. By contrast, H4 Env lost the majority of its activity by 36 hours and showed only 10% activity at the 96-hour timepoint (Fig 3C). Together, these data were consistent with the V3-loop swap energetically stabilizing a closed conformation with a suboptimal CD4-binding site configuration.

To examine how this energetic change altered CD4 utilization during HIV-1 entry, we measured viral infectivity as we titrated available CD4 levels on target cells using the CD4-antagonist DARPin 23.2 (D23.2) [76]. Since H4 and H5 Env engage different CoRs whose expression levels and interaction properties could affect CD4 utilization, we simultaneously used CoRAs to titrate surface CXCR4 and CCR5 levels. At all functional CoR levels, H5 Env was greater than 3.5-fold (average 5.1-fold) more sensitive to D23.2 inhibition than H4 Env (Figs 3D and 3E and S4). Moreover, the steepness of each titration along the D23.2 axis was substantially greater for H5 Env (average Hill Coefficient n_H_ = 1.6) than for H4 Env (n_H_ = 0.73) (S4 Fig). In fact, the CD4- and CoR-dependence to H5 Env-mediated entry were almost identical to that for J5 Env (Figs 3F and S4). Together, the data implied that H5 Env requires a higher cellular CD4 concentration and a higher CD4 binding stoichiometry per Env trimer to support entry. The changes in CD4 dependence to neutralization, shedding and entry induced by the V3-loop swap paralleled previously reported differences between primary isolate and laboratory-adapted Envs [22,45,46,77].

### Swapping V3 loops has a variable impact in other Env strains

**Fig 4:**
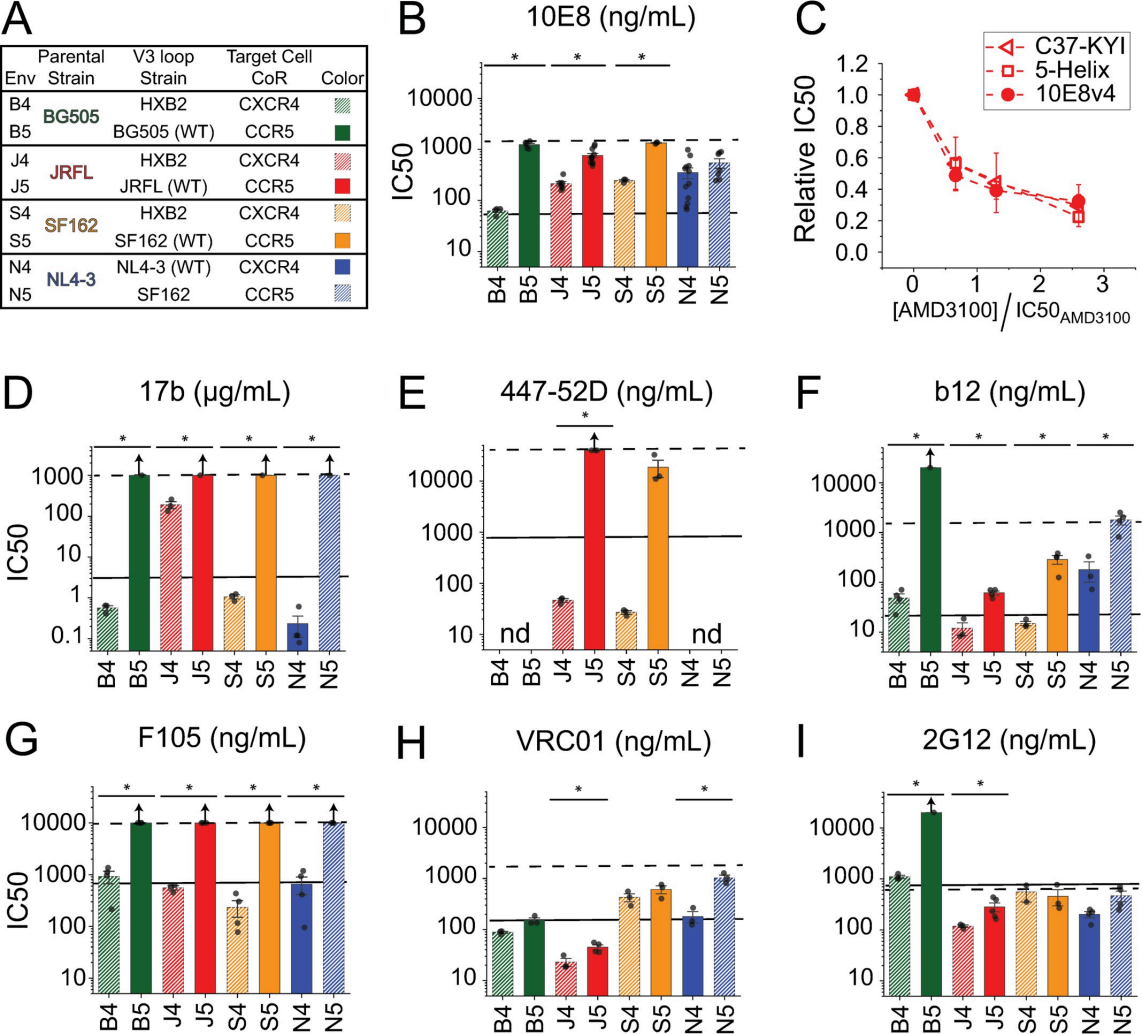
V3-loop swaps have variable effects on epitope exposure in other Env isolates. **(A)** Table listing the V3-loop strains of the tested Env variants. The Env_HXB2_ V3 loop was substituted into Env_BG505_ (green), Env_JRFL_ (red) and Env_SF162_ (orange), while the Env_SF162_ V3 loop was substituted into Env_NL4-3_ (blue). **(B)** IC50 values determined for anti-MPER NAb 10E8 against wild type (solid) and chimeric (hatched) Envs. (**C**) Dependence of C37-KYI, 5-Helix and NAb 10E8 inhibitory potencies on CoRA AMD3100 concentration for J4 Env, the Env_JRFL_ V3-loop chimeric variant. Data are normalized to the IC50 values obtained in the absence of CoRA. Note that CoRA concentration is expressed relative to the AMD3100 IC50 for J4 Env (61 nM). (**D-I**) IC50 values determined for anti-bridging sheet NAb 17b (**D**), anti-V3-crown NAb 447-52D (**E**), anti-CD4 binding site NAbs b12(**F**), F105 (**G**) and VRC01 (**H**), and anti-glycan NAb 2G12 (**I**). In **B** and **D-I**, the solid and dashed black lines indicate the mean IC50 values measured against H4 and H5 Envs, respectively. Bars represent the mean±SEM from three or more independent experiments. Arrows above the bars indicate that 50% neutralization was not achieved at the highest concentrations tested. Asterisks indicate significance as determined by a two-sample t-test (equal variance not assumed) with p < 0.05. In **C**, data points represent the mean±SEM from four independent experiments; there was no significant difference between the three data sets (two-way ANOVA, p > 0.4). In **E**, “nd” denotes not done.

We introduced the HXB2 V3 loop into CCR5-tropic Envs from viral strains JRFL, BG505 and SF162 (Fig 4A). For the JRFL and SF162 strains, the chimeric Envs (hereafter denoted J4 and S4, respectively) adopted CXCR4-tropism and were poorly fusogenic when using CCR5 as CoR (S1 Fig). By contrast, the chimeric Env for the BG505 strain (denoted B4) displayed significant activity using either CXCR4 or CCR5 for viral entry. J4, B4 and S4 Envs were all neutralized more easily by anti-MPER NAb 10E8 compared to their wild type counterparts (Fig 4B and S2 Table). For J4 Env, NAb 10E8 and FI potency showed the same CoRA dependence, suggesting that the higher sensitivity of some chimeric Envs to anti-MPER antibodies did not necessarily correlate with substantially enhanced MPER exposure in the prefusogenic state prior to receptor activation (Fig 4C). In the case of Env_JRFL_, swapping in the Env_HXB2_ V3 loop conferred the same increases in potency to FI C37-KYI and 5-Helix (S5 Fig), suggesting that enhanced NAb 10E8 potency against J4 Env likely resulted from slower transit through the functional intermediate state that exposed the MPER (see Discussion).

The chimeric Envs were also more sensitive to NAbs targeting the CD4i-exposed bridging sheet (17b) and V3-loop crown (447-52D) (Fig 4D and 4E and S2 Table). The V3-loop swaps had a more nuanced effect on the potencies of antibodies targeting the CD4 binding site: NAbs that preferentially bound more activated prefusogenic conformations (b12 and F105) were more potent against chimeric Envs (Fig 4F and 4G), while the NAb that was less sensitive to prefusogenic conformational fluctuations (VRC01) displayed similar potency against the wild type and chimeric species (Fig 4H) [2,78]. As expected, the V3-loop substitution had minimal impact on NAb 2G12 potency with the exception of Env_BG505_ (Fig 4I, the disparity between B4 and B5 sensitivity might reflect a difference in the glycan at residue 295 that rendered B5 Env insensitive to NAb 2G12 [79]). Altogether, introduction of the HXB2 V3 loop appeared to globally increase accessibility to epitopes found in activated Env conformations, similar to the way that mutations in the V1/V2 and V3 loops have been shown to do [48,53,57,60].

We also performed a V3-loop swap in CXCR4-tropic Env_NL4-3_, which is closely related to H4 Env. Inserting the SF162 V3 loop into the NL4-3 background generated the CCR5-tropic chimeric N5 Env (Figs 4A and S1). N5 Env was significantly less sensitive to NAbs b12, F105, VRC01 and 17b, similar to what we had ascertained for H5 Env (Fig 4D and 4F–4H and S2 Table). However, the anticipated decrease in NAb 10E8 potency was not observed (Fig 4B). Qualitatively similar results were found for NAb 2F5 (N4 Env IC50 = 2.3±0.5 μg/ml; N5 Env IC50 = 1.1±0.4 μg/ml), suggesting that accessibility of the entire Env_NL4-3_ MPER was not substantially altered by the Env_NL4-3_-to-Env_SF162_ V3-loop swap. This result was unexpected, especially given the high sequence conservation between Env_NL4-3_ and Env_HXB2_ (97% identity, 22 amino acid differences and a two-residue deletion out of 856 total residues in Env_HXB2_).

### V3-loop residue 306 is an important regulator of MPER exposure in Env_HXB2_ and Env_NL4-3_

**Fig 5:**
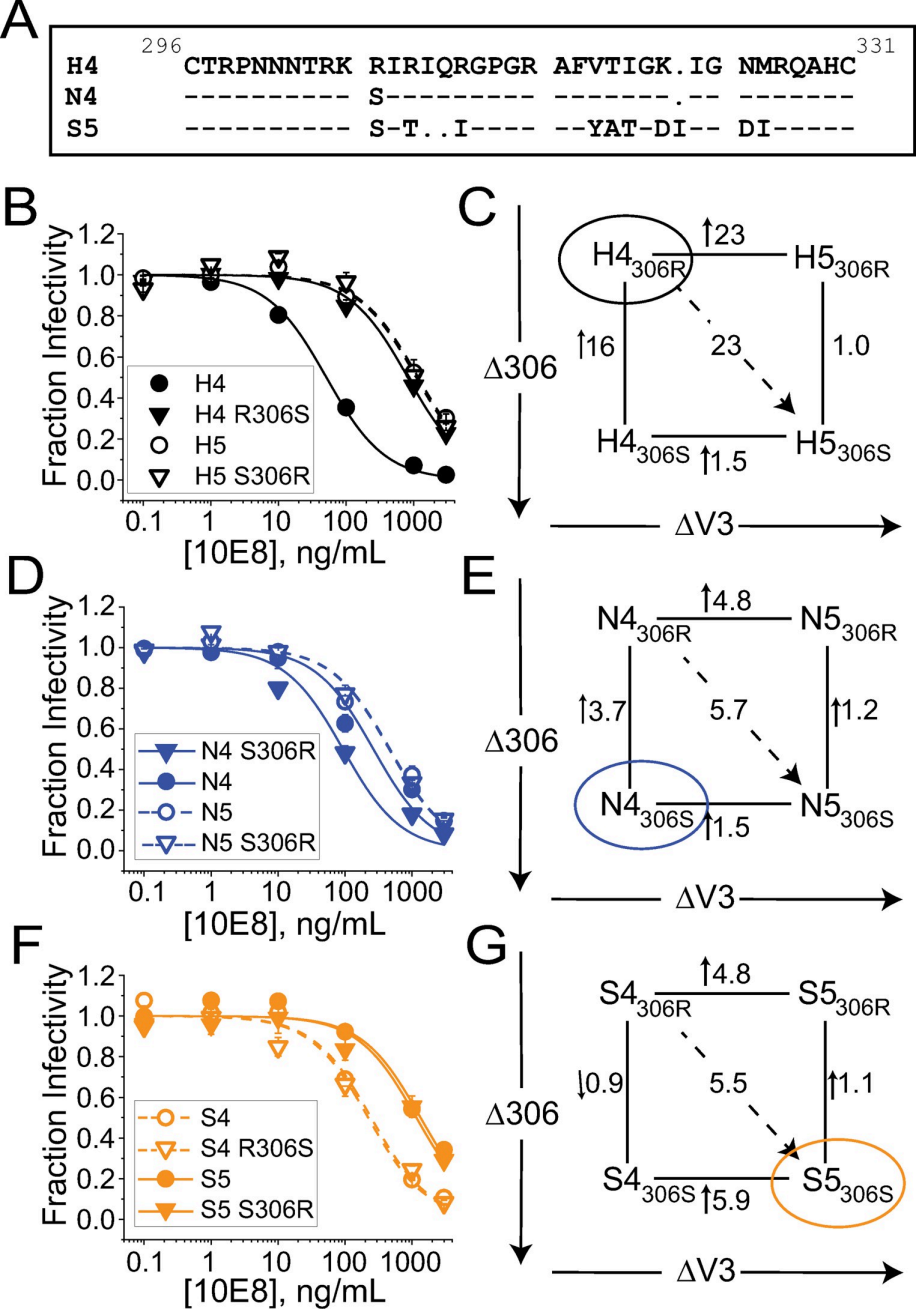
V3-loop residue 306 significantly impacts MPER exposure in Env_HXB2_ and Env_NL4-3_, but not in Env_SF162_. (**A**) Sequence alignment of V3 loops from Env_HXB2_, Env_NL4-3_ and Env_SF162_. (**B**) Inhibitory titrations of NAb 10E8 against Env_HXB2_ variants H4 (filled circles), H4 R306S (filled triangles), H5 (open circles) and H5 S306R (open triangles). (**C**) Box representation of the relative effects that residue 306 substitution (vertical) and V3-loop swap (horizontal) exert on the NAb 10E8 sensitivity of Env_HXB2_. The numbers adjacent to the lines connecting vertical, horizontal and diagonal points indicate the fold-change in IC50 value for the indicated substitution. (**D, E**) As in **B** and **C**, except testing Env_NL4-3_. The wild type Env is designated N4 (filled circles), while the V3-loop chimera is designated N5 (open circles). The S306R substitution was incorporated into both N4 and N5 Envs (filled and open triangles, respectively). (**F, G**) As in **B** and **C**, except testing Env_SF162_. The wild type Env is designated S5 (filled circles), while the V3-loop chimera is designated S4 (open circles). The S306R substitution was incorporated S5 Env (filled triangles), while the R306S substitution was incorporated into S4 Env (open triangles). In each box representation, the wild type Env species is circled.

Using site-directed mutagenesis, we discovered that residue 306 accounted for the differential impact the V3-loop swap had on MPER exposure in Env_HXB2_ and Env_NL4-3_. This residue is an Arg in Env_HXB2_, while it is a Ser in Env_NL4-3_ and Env_SF162_ (Fig 5A). Hence, residue 306 changed identity in the V3-loop swap that produced H5 Env but was preserved in the V3-loop swap that produced N5 Env. In H4 Env, the R306S substitution decreased NAb 10E8 neutralization potency by 16-fold, nearly the entire 23-fold difference between H4 and H5 Envs (Fig 5B). By contrast, the substitution had almost no effect on NAb 10E8 inhibition of H5 Env. The box representation in Fig 5C (see legend) shows that NAb 10E8 resistance was achieved by either a V3-loop swap that maintained Arg at position 306 (top horizontal) or by an R306S substitution in the HXB2 V3-loop background (left vertical). Neither a V3-loop swap with Ser at position 306 (bottom horizontal) nor an R306S mutation in the SF162 V3-loop background (right vertical) significantly impacted NAb 10E8 potency. Together, the results implied that the R306S substitution worked in concert with the remainder of the HXB2 V3 loop to modify MPER accessibility, an effect that was blunted in context of the SF162 V3 loop (see Discussion).

Substituting Ser306 with an Arg enhanced N4 Env sensitivity to NAb 10E8 almost 4-fold, but had minimal impact on N5 Env sensitivity (Fig 5D and S2 Table). In the box representation of Fig 5E, the diagonal represents the equivalent of the H4-to-H5 Env transformation: in the Env_NL4-3_ background, that transition results in a 5.7-fold increase in NAb 10E8 IC50. Again, major changes to NAb 10E8 potency occur along the top horizontal and left vertical, with the other transitions having only minor ramifications. The result matched what we observed for Env_HXB2_, although the differences were quantitatively smaller. In contrast to the CXCR4-tropic Envs, the point mutation at residue 306 exerted no effect on antibody neutralization of either S4 or S5 Envs (Fig 5F and 5G and S2 Table). In other words, the increased NAb 10E8 potency against S4 Env occurred independent of the amino acid at residue 306. The same phenotype was observed in the Env_JRFL_ background (S2 Table). The results implied that differences from the H4 and N4 Env sequences outside the V3 loop enabled S4 and J4 Envs to weather an Arg residue at position 306.

### The R306S mutation destabilizes the MPER-accessible prefusogenic conformation of Env_HXB2_

**Fig 6:**
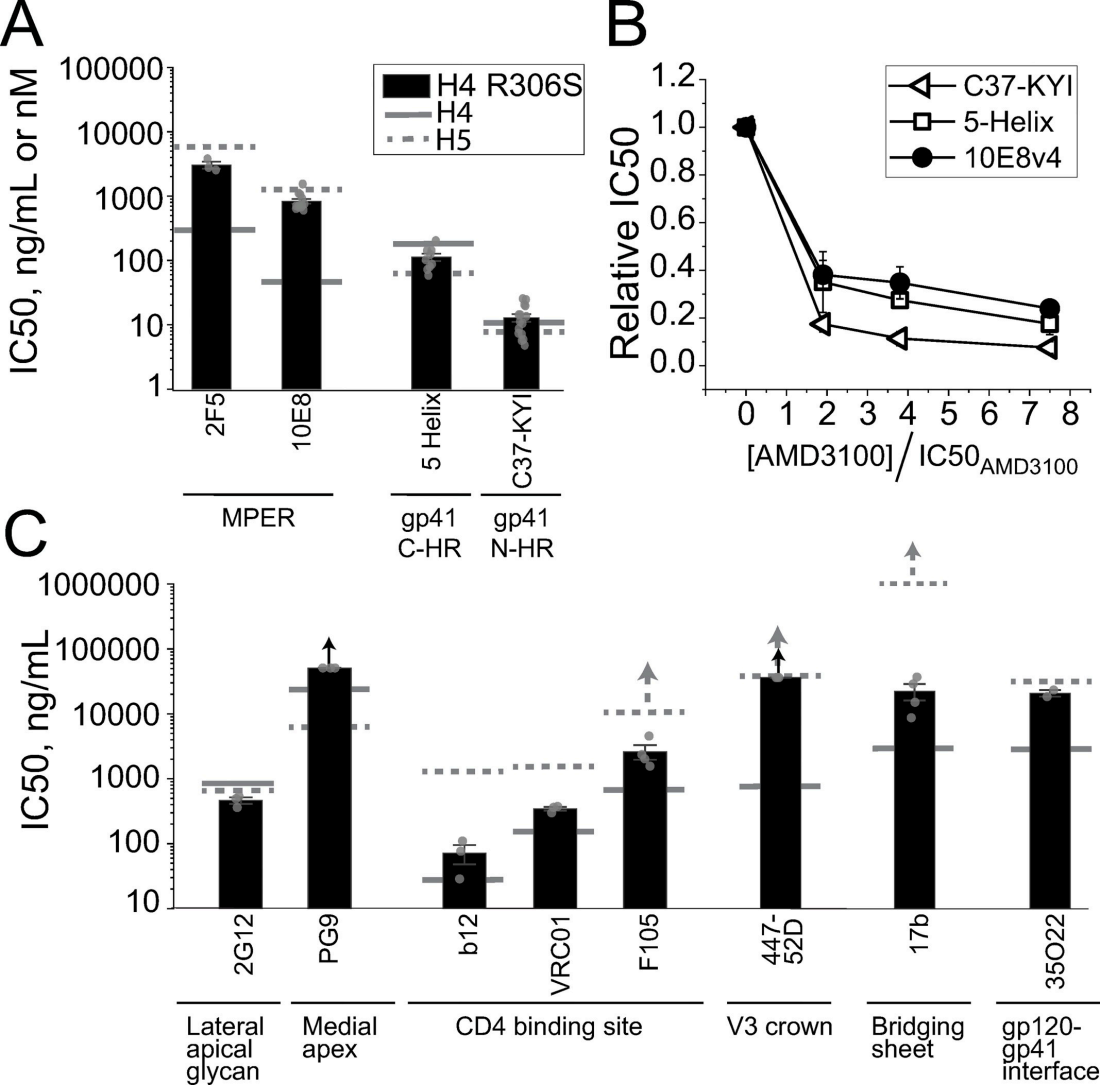
H4 R306S Env has an antibody sensitivity profile distinct from both wild type H4 Env and the chimeric H5 Env. (**A**) Anti-MPER antibody (2F5, 10E8) and FI (5-Helix, C37-KYI) neutralization potencies against H4 R306S Env. The solid and dashed gray lines reflect the average IC50 values obtained for H4 and H5 Envs, respectively. NAbs 2F5 and 10E8 were significantly less potent against H4 R306S Env than against wild type H4 Env; the antibody potencies against H4 R306S Env and H5 Env were not significantly different (one-way ANOVA with means comparison using the Tukey method, p < 0.025 for H4 / H4 R306S comparison [109]). (**B**) Impact of CoRA AMD3100 on the inhibitory potencies of C37-KYI (triangles), 5-Helix (squares) and NAb 10E8v4 (circles) against HIV-1 pseudotyped with H4 R306S Env. Data are normalized to the IC50 values obtained in the absence of CoRA. Note that CoRA concentration is expressed relative to the AMD3100 IC50 for H4 R306S Env (53 nM). The data sets for 5-Helix and 10E8v4 are not statistically different as assessed by two-way ANOVA (p = 0.9); the difference in data sets for C37-KYI and 10E8v4 does achieve significance (p = 0.022). (**C**) Anti-gp120 and anti-interface antibody neutralization potencies against H4 R306S Env. Arrows above the bars and dotted lines indicate that 50% neutralization was not achieved for H4 R306S and H5 Envs at the highest concentrations tested. Of these antibodies, only NAbs 447-52D, 35O22 and PG9 had significantly lower potencies in comparisons of H4 R306S and H4 Env (one-way ANOVA with means comparison using the Tukey method, p < 0.025). All data reflect the mean±SEM of three or more independent experiments.

The R306S substitution in H4 Env disrupted neutralization by anti-MPER NAbs 2F5 and 10E8 to the same extent without altering FI activity (Fig 6A). The loss of antibody potency was not accompanied by an appreciable loss of binding affinity, as antibody neutralization remained irreversible (S2 Fig). Moreover, the point mutation altered the CoRA-dependence of NAb 10E8 neutralization, which now matched that for FI 5-Helix inhibition (Fig 6B). The results suggested that the R306S substitution largely restricted anti-MPER antibody binding to a functional intermediate state, as had occurred with the V3-loop swap that produced H5 Env.

While the V3-loop swap imparted relative resistance to antibodies targeting epitopes exposed in more-activated Env conformations, the R306S substitution had a more varied impact (Fig 6C). Like H5 Env, H4 R306S Env was significantly less sensitive to antibodies targeting the V3-loop crown (447-52D) and gp120-gp41 interface (35O22). However, H4 R306S Env remained mostly sensitive to antibodies targeting the CD4 binding site and CD4i-exposed bridging sheet. Moreover, the potency of NAb PG9, which preferentially binds the fully closed, apo-conformation of Env, was not enhanced by the R306S substitution, as it was by the V3-loop swap. These results indicated that the R306S substitution and V3-loop swap had different effects on the energy landscape of prefusogenic Env conformational fluctuations.

**Fig 7:**
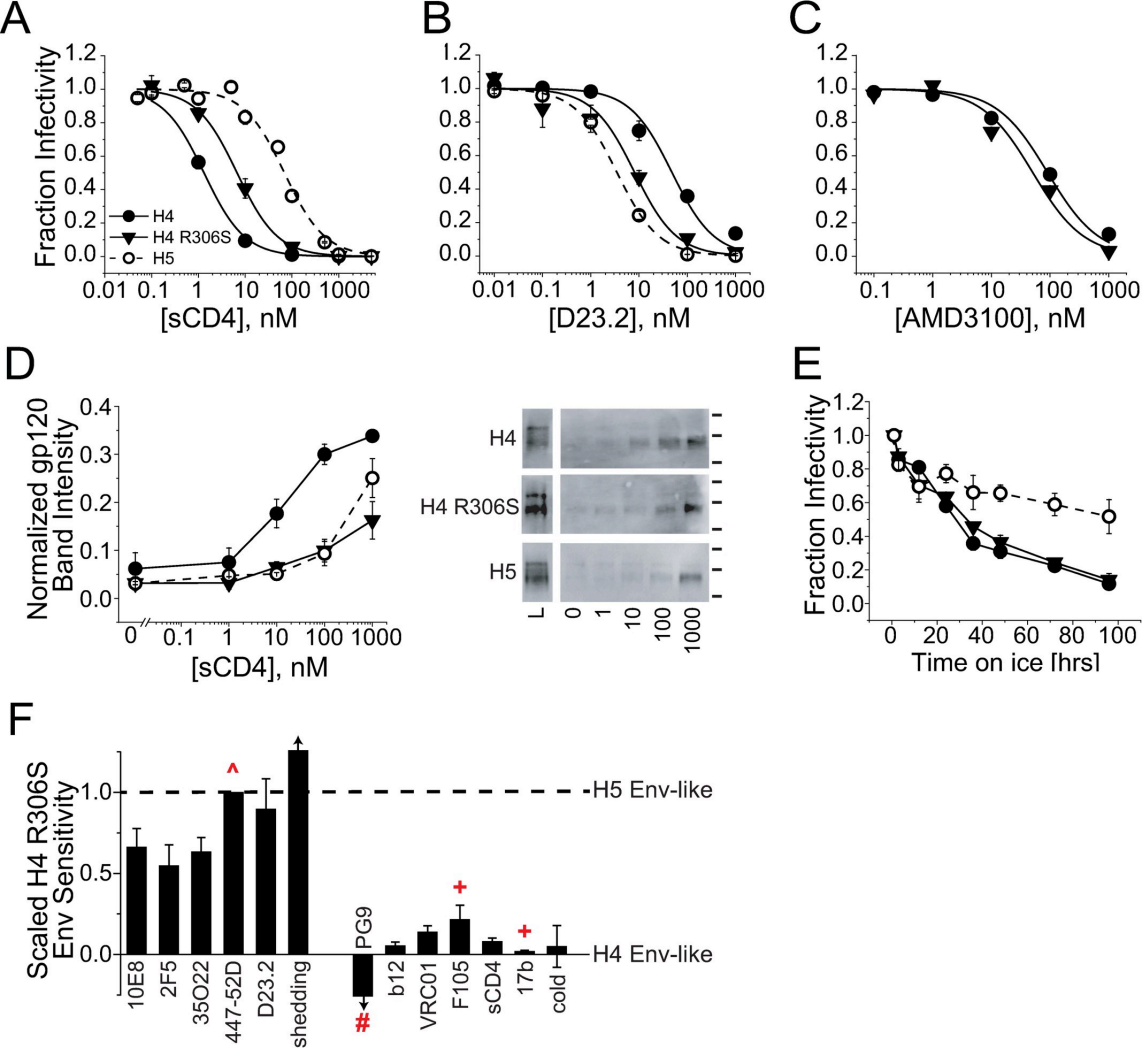
The R306S substitution in H4 Env conferred some of the functional and energetic properties of H5 Env while maintaining others from the H4 Env background. (**A-C**) Inhibitory titrations of sCD4 (**A**), CD4 antagonist D23.2 (**B**) and CoRA AMD3100 (**C**) against HIV-1 pseudotyped with H4 Env (filled circles), H5 Env (open circles) and H4 R306S Env (triangles). Of these three inhibitors, only D23.2 showed significantly different potencies when comparing H4 R306S to H4 Env (one-way ANOVA with means comparison using the Tukey method, p < 0.025). (**D**) Soluble CD4 dependence to gp120 shedding from H4, H4 R306S and H5 Env-expressing cells. Shed gp120 was detected by SDS-PAGE/Western blot, quantified by densitometry and normalized to the total gp120 detected in cell lysates. In the images of representative Western blots (right), L denotes a 1:3 dilution of cell lysate; the lines to the right of the images indicate positions of 250, 150 and 100 kDa molecular weight markers. The H4 R306S Env data set is significantly different from the H4 Env data set (two-way ANOVA, p < 0.01) but not statistically different from the H5 Env data set (p > 0.20). (**E**) Time dependence to cold inactivation of HIV-1 pseudotyped with H4, H4 R306S or H5 Env. The H4 R306S data set is significantly different from the H5 Env data set (two-way ANOVA, p < 0.01) but not statistically different from the H4 Env data set (p > 0.40). Data points in **A-E** represent the mean±SEM of three or more independent experiments. (**F**) Sensitivity profile of H4 R306S Env scaled to the differences between H4 and H5 Env sensitivities. For each inhibitor, the IC50 value for H4 R306S Env was transformed according to the following formula: $Scaled\;IC50=\frac{{IC50}_{H4\;R306S}-{IC50}_{H4}}{{IC50}_{H5}-{IC50}_{H4}}$ For gp120 shedding (“shedding”), the sCD4 concentrations that elicited a normalized band intensity of 0.16 (estimated from the data lines in **D**) were used in place of IC50 values in the equation above. For cold denaturation (“cold”), the infectivity fractions at 96 hours were used in place of IC50 values. The scaled value provides the relative similarity of H4 R306S Env to either H4 Env (scaled sensitivity = 0) or H5 Env (scaled sensitivity = 1, dotted line). Data in **F** were calculated using average IC50 (or other) values with errors formally propagated. Red symbols designate that the IC50 value for H5 Env (+), H4 R306S Env (#) or both (“^”) exceeded what we could measure. In these situations, the scaled value shown is an overestimate (+), underestimate (#) or merely an estimate (^) of the true value.

The varied impact of the R306S substitution also extended to functional characteristics regarding CD4 binding versus utilization for entry. The sensitivity of H4 R306S Env to sCD4 inhibition was much closer to that of wild type H4 Env than H5 Env (Fig 7A), suggesting that binding was not particularly affected by the point mutation. By contrast, the sensitivity of H4 R306S Env to CD4-antagonist D23.2 more closely resembled that for H5 Env than H4 Env (Fig 7B), indicating that the point mutation had a significant impact on CD4 utilization during entry. This shift in CD4 utilization was not accompanied by a significant change in CoR utilization, as wild type and mutant H4 Envs were similarly sensitive to CoRA AMD3100 (Fig 7C). Thus, despite only minimally perturbing CD4 interaction with the Env trimer, the R306S substitution dramatically altered CD4 binding requirements for viral entry.

This dichotomy in structural and functional consequences of the R306S substitution was also reflected in the results from Env-stability measurements. The R306S variant had lower spontaneous gp120 shedding than wild type Env and was relatively resistant to sCD4-induced shedding; in fact, the data closely mimicked that for the stable H5 Env variant (Fig 7D). Surprisingly, the R306S substitution had no appreciable impact on cold inactivation, with the mutant variant sensitivity overlapping that for the relatively unstable wild-type H4 Env (Fig 7E). These observations illuminated the different energy landscapes of H4, H4 R306S and H5 Envs and, additionally, showed that the two measures of Env stability report on independent biochemical processes.

To summarize, H4 R306S Env adopted some characteristics of H5 Env while retaining properties of its H4 Env background (Fig 7F). Specifically, the R306S variant acquired resistance to antibodies targeting the MPER, gp120-gp41 interface and V3-loop crown, increased its requirement for CD4 to promote viral entry, and decreased its sensitivity to sCD4-induced shedding. By contrast, H4 R306S Env maintained (in)sensitivity to antibodies targeting several gp120 conformational epitopes, including the V1/V2 loop, CD4 binding site and CD4i-exposed bridging sheet. In addition, the point mutation only minimally perturbed CD4 binding affinity and sensitivity to cold denaturation. These changes were quite distinct from the impact of the V3-loop swap, which appeared to stabilize the glycoprotein trimer in its closed conformation and to increase the CD4 requirement for activation. The sensitivity of H4 R306S Env to antibodies targeting activated conformations (e.g., F105, 17b) implied that the mutant trimer could still readily fluctuate into open structures, but not to ones that allowed easy access to gp41 epitopes. Curiously, exposure of the V3 crown in H4 R306S Env appeared to occur in these later MPER-accessible states, even though earlier fluctuations effectively exposed the bridging sheet that lies underneath the V3 loop.

### Towards a mechanism of how residue 306 modulates MPER accessibility

**Fig 8:**
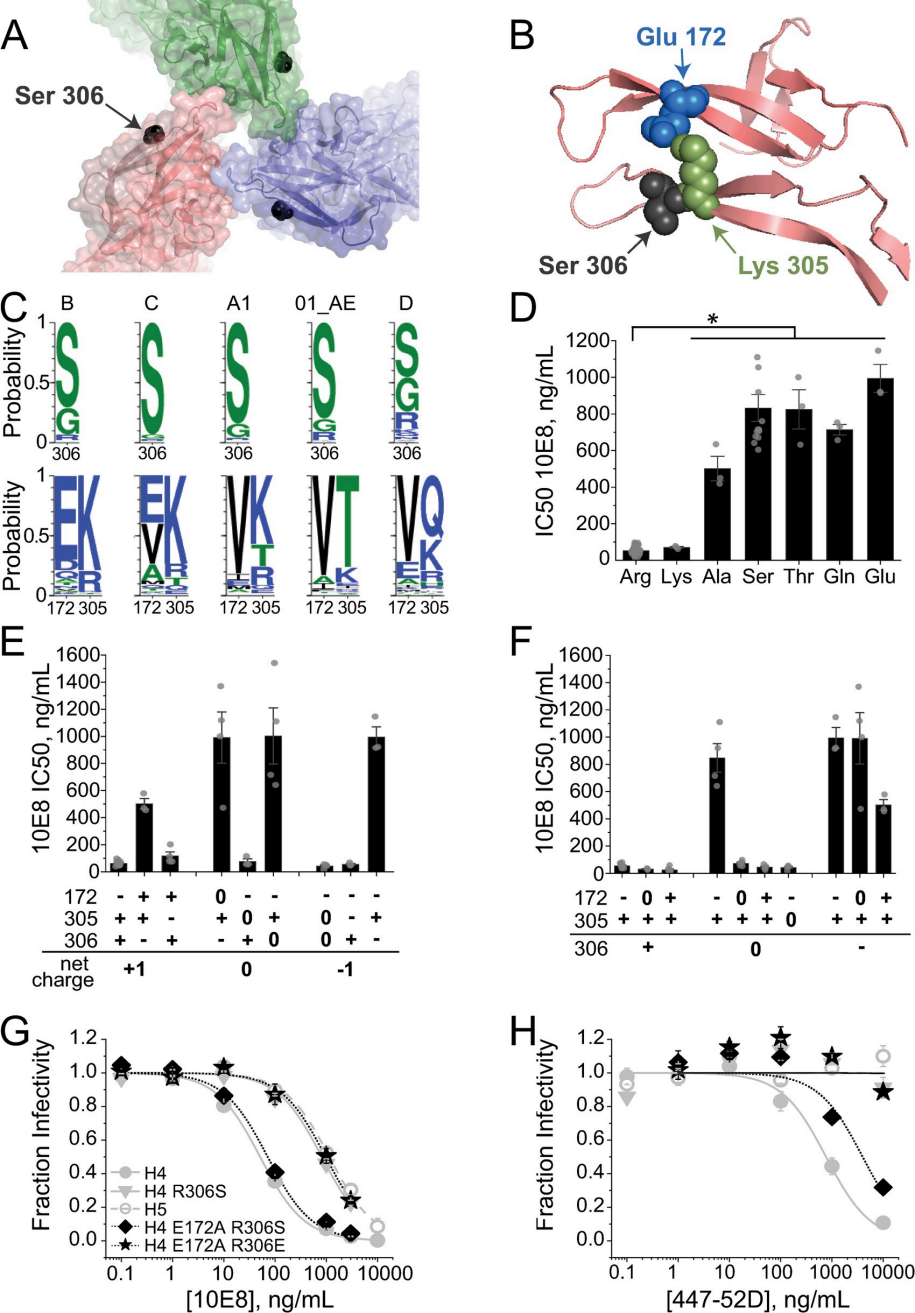
The charge at V3-loop residue 306 regulates gp41 MPER accessibility in H4 Env through short-range intrasubunit interactions. (**A**) Surface and ribbon rendering of the apical structure of Env_JRFL_ SOSIP.664 (PDB 5FYK [79]) showing the position of residue 306 (Ser, black). (**B**) Ribbon diagram of the V1/V2 and V3 loops from one gp120 subunit highlighting V1/V2-loop residue Glu172 (blue) and V3-loop residues Lys305 (green) and Ser306 (black). (**C**) Logos plots showing residue conservation at position 306 (top) and positions 172 and 305 (bottom) across different HIV-1 clades. Plots were obtained from the LANL HIV Sequence Database using the Analyze Align tool. (**D**) NAb 10E8 potency against HIV-1 pseudotyped with mutant H4 Env variants containing substitutions at Arg306. The asterisk indicates that the 10E8 IC50 values of all H4 Env variants except the R306K mutant are significantly higher than that for wild type H4 Env (one-way ANOVA with means comparisons using the Tukey method, p < 0.001 [109]). (**E**, **F**) NAb 10E8 potency against HIV-1 pseudotyped with mutant H4 Env variants containing charge substitutions at triad residues Glu172, Lys305 and Arg306. Amino acids corresponding to the “-“, “0” and “+” designations along the x-axis are as follows: Glu (-), Ala (0) and Lys (+) for residues 172 and 305; Glu (-), Ser (0) and Arg (+) for residue 306. In **E**, mutant Envs are grouped according to the total triad charge. In **F**, mutant Envs are grouped according to the charge at residue 306. (**G, H**) Inhibitory titrations of NAbs 10E8 (**G**) and 447-52D (**H**) against wild type H4 Env (gray filled circles) and variants H4 R306S (gray triangles), H5 (gray open circles), E172A/R306S (black diamonds) and E172A/R306E (black stars). Data bars and points represent the mean±SEM of three or more independent experiments, except for NAb 447-52D titrations of H4 E172A/R306S and H4 E172A/R306E Envs in **H**, where the data reflect the mean±range of mean of two independent experiments.

Currently, only CCR5-tropic Envs with a Ser at position 306 have been structurally characterized to high resolution. Prior to CD4 activation, the amino acid is located on the lateral face of the V3 loop exposed to aqueous solution between gp120 subunits (Fig 8A and 8B). Ser306 abuts the side chain of Tyr318 from the opposite strand of the V3 loop but does not appear to make any other substantial interactions (S6A Fig). Ser in position 306 is conserved in 79.5% of sequenced HIV-1 Envs (Los Alamos National Laboratories HIV Sequence Database, January 2022, 6223 total Envs), but conservation significantly varies by clade (Figs 8C and S6B). Arg or Lys is found at that position with an overall frequency of 4.11%, but, again, this number shows high variability across clades (Figs 8C and S6B).

When Arg306 was substituted with a Lys in H4 Env, the IC50 value for NAb 10E8 remained low (Fig 8D). By contrast, when Arg306 was replaced with either a neutral or acidic amino acid, NAb 10E8 IC50 values increased 9.5-fold or more, with the R306E variant showing the largest change (19-fold). These changes in NAb 10E8 sensitivity were independent of the side chain at residue 318, indicating that an interaction between residues 306 and 318 does not determine MPER exposure (S6C Fig). Based on these results and on the location of Ser306 in available Env structures, we hypothesized that the residue at position 306 exerted its effects on Env conformational equilibrium via through-space electrostatic interactions. To test this idea, we introduced charge-altering mutations at Arg306 and two spatially adjacent charged residues, Glu172 and Lys305 (Fig 8B). We reasoned that NAb 10E8 potency should track with net charge on this amino-acid triad if control of MPER exposure were mediated by long-range electrostatics that contributed to subunit-subunit interactions. Wild-type H4 Env has a net triad charge of +1 and high sensitivity to NAb 10E8 neutralization. However, the configuration of the individual charges could be rearranged to produce an Env variant with a triad charge of +1 that was relatively resistant to NAb 10E8 inhibition (Fig 8E). Similarly, Envs with net triad charges of 0 or -1 manifested both low and high NAb 10E8 sensitivity. These observations were inconsistent with long-range electrostatics between subunits governing MPER accessibility.

The dependence of NAb 10E8 sensitivity on triad charge configuration suggested that MPER exposure could depend on intra-subunit interactions mediated by the three apical residues. In the prefusogenic structure of clade B Env_JRFL_ [79], the side chains of Glu172 and Lys305 appear to form an ionic bond that bridges the V1/V2 and V3 loops (Fig 8B). Of sequenced clade B isolate Envs, almost 78% have an acidic amino acid at position 172 and a basic amino acid at position 305 (Figs 8C and S6B). We systematically evaluated the role of this putative ionic bond in H4 Env by substituting Glu172 with either an Ala or a Lys in the context of an Arg, Ser or Glu at residue 306. The ionic interaction did not play a role in MPER exposure when residue 306 was charged: NAb 10E8 inhibited all Arg306 variants with high potency and all Glu306 variants with low potency (Fig 8F). However, in context of Ser at position 306, MPER occlusion was only observed with an intact 172–305 ionic bond, while Env_HXB2_ variants with a E172A, E172K, or K305A mutation were highly susceptible to NAb 10E8 neutralization (Fig 8F).

The observations above suggested that an electrostatic interaction between V1/V2 and V3 loops somehow controlled exposure of the MPER epitope. Such an interaction could potentially restrict access to the V3-loop crown and explain the correlation in potencies of antibodies targeting these spatially disparate epitopes. To explore this possibility, we compared the impact of disrupting the putative 172–305 ionic bond on the potencies of NAbs 10E8 and 447-52D. In the context of a Ser at position 306, the E172A mutation significantly enhanced susceptibility to both NAb 10E8 and NAb 447-52D neutralization (Fig 8G and 8H). By contrast, the E172A mutation exerted no effect on the low neutralization potency of either antibody when position 306 was Glu. The results supported an electrostatic interaction between V1/V2 and V3 loops that governed exposure of both the V3-loop crown and MPER epitope in Env_HXB2_.

The potential for an ionic bond between residues 172–305 is not well conserved across HIV-1 clades (S6D Fig). However, the two positions show a high degree of coevolution, indicating energetic coupling between the amino acids [80]. Interestingly, in Env_BG505_, which lacks an acidic residue at position 172, Lys305 appears to make a cation-π interaction with aromatic V2-loop reside Tyr173 (S6E Fig). Disruptions to this interaction have been shown to increase susceptibility to anti-gp120 antibody neutralization in several Env strains [61]. In fact, aromatic residues are highly conserved at position 173, which also strongly coevolves with position 305 [80]. The potential for this cation-π interaction remains high in most HIV-1 clades (S6F Fig). These findings suggest that an interaction between V1/V2 and V3 loops adjacent to position 306 is a common feature of Env. Based on our observations in H4 Env, we speculate that the charge on residue 306 modulates the strength of this loop-loop interaction, which, in turn, determines the significance of disrupting the 172–305 ionic bond or 173–305 cation-π interaction in regulating MPER accessibility (see Discussion).

## Discussion

As one of two major regions involved in interprotomer interactions, the apex of HIV-1 Env has long been recognized as a key regulator of prefusogenic glycoprotein structure and dynamics. Destabilizing mutations in the V1/V2 and V3 loops of primary isolate Envs have been shown before to enhance exposure of neutralizing epitopes in both gp120 and gp41 [48,53,57–62]. In this study, we have accomplished the reverse in whole or in part: swapping the Env_SF162_ V3 loop into the laboratory-adapted isolate Env_HXB2_ bestowed tier 2-like, primary isolate neutralization sensitivity to H5 Env, while introducing a point mutation at position 306 in the Env_HXB2_ V3 loop selectively conferred resistance to NAbs targeting the gp120 V3-loop crown and gp41 MPER. The apparent structural disparity in H4, H4 R306S and H5 Envs implies differential stabilization of states along the activation pathway that governs prefusogenic Env dynamics.

Historically, the overall stability of HIV-1 Env has been characterized through temperature-induced inactivation and gp120 shedding. Typically, the two measures show a high degree of correlation: Envs with low sensitivity to cold- or hot-induced inactivation are most often resistant to gp120 shedding, both spontaneous and CD4-induced [22,43,44,47,55,56]. Such Envs are relatively insensitive to broad spectrum antibody neutralization, require higher levels of cellular CD4 to trigger membrane fusion and mediate very little CD4-independent entry [22,39,43,45,46]. These properties are thought to reflect the high stability of a closed apo-structure that energetically limits fluctuations along the activation pathway in the absence of receptor binding [35,36,41]. High stability is a characteristic feature of Envs from primary isolate HIV-1 strains, likely selected in order to limit neutralizing immune responses.

Laboratory adaptation of HIV-1 results in Envs of lower stability with less-pronounced energetic differences between apo- and receptor-induced conformations [35]. The resulting increase in receptor-independent conformational fluctuations along the activation pathway leads to enhanced exposure of neutralizing epitopes, increased sensitivity to temperature-induced inactivation, and decreased requirement of CD4 to trigger viral entry or gp120 shedding [21,39,43,45]. These characteristics well describe the properties of the laboratory-adapted clade B isolate H4 Env, which has considerable sequence divergence from clinical clade B primary isolates (86% identity/5 gaps with Env_JRFL_; 89% identity/7 gaps with clade B Env consensus sequence, Los Alamos National Laboratory HIV Sequence Database). However, the properties of H5 Env imply that laboratory adaptation has not fundamentally altered the physiochemical properties of Env_HXB2_: in addition to its tier 2-like sensitivity to antibody and sCD4 neutralization, H5 Env displays characteristics of high stability Envs including resistance to both sCD4-induced gp120 shedding and cold inactivation and a requirement for high levels of CD4 on target cells for entry. Interestingly, the single point mutation R306S in the H4 Env V3 loop confers partial stability to the glycoprotein trimer. Like H5 and J5 Envs, H4 R306S Env is resistant to sCD4-induced gp120 shedding and requires high levels of CD4 on target cells for entry. However, H4 R306S Env has the same sensitivity to cold inactivation as wild type H4 Env. These results indicate that the two measures of Env stability reflect different elements of the prefusogenic energy landscape.

**Fig 9:**
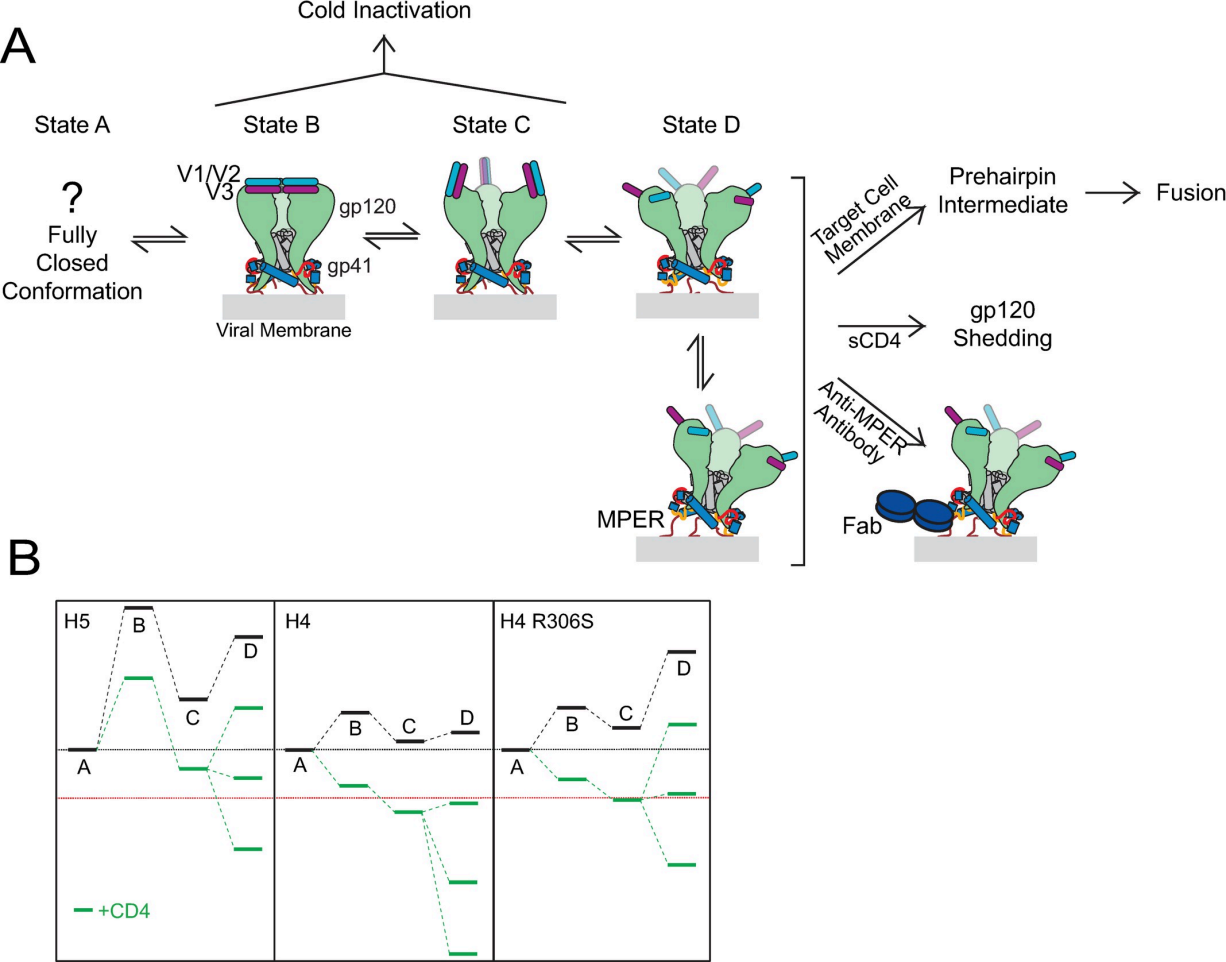
The V3-loop swap and R306S mutation have differential effects on the conformational equilibrium of Env_HXB2_. (**A**) Model of HIV-1 Env conformational fluctuations in the prefusogenic state. The gp120 trimer (green) adopts a closed conformation in which the apical regions mediate interprotomer interactions (States A and B). Many neutralizing epitopes, including the gp41 MPER at the trimer base, are occluded in these states. Relaxation of the gp120 subunits leads to loss of apical interprotomer interactions, causing the V1/V2 (light blue) and V3 (magenta) loops to flip up in tandem, exposing numerous underlying epitopes, including the gp120 bridging sheet (state C). Finally, the V1/V2 and V3 loops splay, increasing trimer flexibility and enhancing its propensity to tilt relative to the membrane, thereby sufficiently exposing the gp41 MPER for antibody binding (Fab shown in dark blue) [82]. Note that only a single Fab binding is shown, even though there could be up to three antibodies bound per trimer. When sufficiently stabilized by CD4 binding, the fully-open State D conformation can be triggered by CoR interactions to complete conformational transitions that promote membrane fusion. Sensitivity to cold inactivation appears to reflect the ability of Env to spontaneously fluctuate into states B and C, while the CD4-dependence to gp120 shedding appears to correlate with the stability of state D. (B) Proposed impact of the V3-loop swap and R306S mutation on prefusogenic state energy levels in Env_HXB2_. Black lines reflect unbound conformations, while green lines reflect CD4-bound states (for clarity, binding stabilization by more than one CD4 is shown only for state D). The red dotted line indicates the stabilization required to shift conformational equilibrium sufficiently into State D for efficient CoR-triggering of downstream structural transitions. The V3-loop swap that produced H5 Env primarily stabilizes state A relative to states B, C and D, shifting the equilibrium away from the more epitope-accessible conformations. Consequently, overall sensitivity to antibody neutralization, susceptibility to cold inactivation, and vulnerability to sCD4-induced gp120 shedding are reduced. By contrast, the R306S substitution stabilizes states A, B and C (states with interacting V1/V2 and V3 loops) relative to state D. As a result, sensitivity to anti-gp120 MPER antibodies and to sCD4-induced shedding is reduced, while sensitivity to anti-gp120 antibodies and to cold inactivation is largely unaffected. Both alterations increase the CD4 binding stoichiometry required to achieve sufficient stabilization of state D for CoR-triggered membrane fusion, accounting for the increased sensitivity of H5 and H4 R306S Envs to CD4 antagonist D23.2.

Despite its resistance to antibodies targeting the V3-loop crown and MPER, H4 R306S Env remains sensitive to antibodies that selectively bind activated Env conformations, including the bridging sheet that lies underneath the V3 loop. Differential exposure of these epitopes necessitates at least two post-CD4 binding Env conformations. Comparison of the neutralization sensitivities of H4, H4 R306S and H5 Env implies that the V3-loop crown / MPER accessible conformation is downstream of gp120 fluctuations that expose the bridging sheet. Given the temporospatial properties of MPER exposure, we conclude that these two states lie on the fusion pathway prior to the transition into the PHI. Finally, based on our manipulations of the charged triad at the Env_HXB2_ apex, we posit that fluctuation into the MPER accessible state is tied to the loss of an interaction between V1/V2 and V3 loops regulated by the charge at position 306 and supported by an ionic bond between V1/V2 residue 172 and V3 residue 305. These considerations lead us to propose a new state along the activation pathway in which the V1/V2 and V3 loops disengage from the gp120 core, remaining associated with each other but abrogating intersubunit interactions with loops on adjacent protomers (State C in Fig 9). This partially opened conformation exposes previously occluded gp120 regions (e.g., the bridging sheet) while still shielding the V3-loop crown and MPER. Ultimately, the trimer transitions into its fully open, V3-loop crown and MPER-accessible conformation through splaying of the V1/V2 and V3 loops on either side of the gp120 subunit as seen in structures of CD4-17b-gp120/gp41 and CD4-CCR5-gp120 [11–13,18] (State D in Fig 9). How V1/V2- and V3-loop interactions are tied to MPER occlusion is currently unknown, but the splaying might increase Env flexibility, enhancing the ability of the trimer to tilt relative to the membrane (depicted in Fig 9) as observed in recent cryo-EM structures of Env trimers in viral membranes [81] and in artificial lipid bilayers bound to a single 10E8 antibody [82].

As a working model, we consider the 4-state Env-activation pathway depicted in Fig 9. A fully closed, unliganded conformation (state A, heretofore not structurally elucidated) transitions into the closed state depicted in many SOSIP structures (state B) after a single CD4 binding event [36,37,83,84]. Binding of multiple CD4s induces transition first into State C, with the V1/V2 and V3 loops flipped up in tandem, and then into State D, with the V1/V2 and V3 loops splayed. Many anti-gp120 antibodies that bind activated conformations (e.g., NAbs F105 and 17b) can interact with Env in states C and D, while anti-V3-crown and anti-MPER antibodies can only bind to state D.

We predict that state A is much more stable than the downstream states in H5 Env, as it appears to be in tier 2/3 primary isolates (Fig 9B) [35–37,47]. Effective transition for such Envs into states C and D would require stabilization from CD4 binding, causing the bridging sheet and MPER epitopes to be exposed only transiently during membrane fusion. Consequently, neutralization by NAbs 17b, 447-52D and 10E8 is kinetically restricted like bona fide PHI fusion inhibitors, accounting for the relatively poor potencies for these antibodies and the strict CoRA-dependence to NAb 10E8 inhibitory activity. By contrast, the energy differences between state A and downstream states in H4 Env are considerably lower, enabling frequent CD4-independent fluctuations into states C and D (Fig 9B). As a result, NAbs 17b, 447-52D and 10E8 have a chance to engage Env outside the context of target-cell interaction, thereby increasing their neutralization potencies and decreasing the CoRA-dependence to NAb 10E8 inhibitory activity. In our model, the R306S mutation in H4 Env primarily impacts the energy difference between states C and D (when bonds between the V1/V2 and V3 loops are broken), leaving the energy differences between states A, B and C largely unchanged (Fig 9B). Because CD4 binding is required to sufficiently expose the gp41 MPER, NAb 10E8 neutralization of H4 R306S Env becomes kinetically restricted (H5 Env-like) while neutralization by anti-gp120 antibodies, including 17b, is mostly unaltered (H4-Env like).

Our analysis clarifies why the CD4-dependence to viral entry (including Hill coefficient, S4 Fig) correlates with gp120 shedding but not necessarily with cold inactivation. According to our model, both H5 and H4 R306S Envs require higher CD4 binding stoichiometry compared to H4 Env in order to achieve the stabilization of state D needed for efficient CoR-triggered irreversible transition into the PHI (red line in Fig 9B). Our data suggest that gp120 shedding reports on this required CD4 binding stoichiometry. By contrast, cold inactivation seems to reflect the stability of state A relative to states B and C. Thus, CD4-induced gp120 shedding appears to follow on-pathway conformational changes through (at least) state D, whereas cold inactivation diverges from the activation pathway somewhere between states A and C.

Our results indicate that the Env_HXB2_ V3 loop is primarily responsible for the laboratory-adapted phenotype of H4 Env. The interprotomer electrostatic repulsion resulting from the additional six positive charges per V3 loop (compared to the Env_SF162_ V3 loop) is likely very destabilizing, especially since these cations are concentrated in the gp120-gp120 interface at the apex of Env. However, introduction of the Env_HXB2_ V3 loop into Env_JRFL_ had only a modest effect on antibody neutralization and no effect on the CoRA-dependence to MPER exposure. It is clear that Env_JRFL_ regions outside of the V3 loop function to blunt the impact of apical destabilization. Curiously, swapping the Env_HXB2_ V3 loop into Env_BG505_ did produce a substantial increase in global epitope exposure that rivaled and even exceeded changes observed for the V3-loop swap in Env_HXB2_ (with the notable exception of NAb VRC01, which has the ability to interact with the CD4-binding site in multiple states [78]). Ongoing work is focused on dissecting the differences between primary isolate Envs that make them more or less susceptible to apical disruptions. Our attention here on Env_HXB2_, however, highlights the utility of using less stable laboratory-adapted isolates in parsing out more nuanced aspects of Env conformational dynamics that might not be as easily elucidated in more stable primary isolate trimers.

## Materials and methods

### Cell lines and reagents

The following cell lines and reagents were obtained from NIH HIV Reagent Program, Division of AIDS, NIAID, NIH:

Cell lines: U87 CD4+CCR5+ cells (ARP-4035) and U87 CD4+CXCR4+ cells (ARP-4036) contributed by Drs. Hong Kui Deng and Dan Littman [85]

Anti-Human Immunodeficiency Virus (HIV)-1 monoclonal antibodies: 2F5 (ARP-1475) contributed by DAIDS/NIAD [86]; 4E10 (ARP-10091) contributed by DAIDS/NIAD [87]; 10E8 (ARP-12294) contributed by Dr. Mark Connors [88]; 10E8v4 (ARP-12865) contributed by Dr. Peter Kwong [89]; IgG1 b12 (ARP-2640) contributed by Dr. Dennis Burton and Dr. Carlos Barbas [90]; VRC01, produced in *vitro* (ARP-12033) contributed by Dr. John Mascola [91]; F105 (ARP-857) contributed by Dr. Marshall Posner and Dr. Lisa Cavacini [92]; clone 17b produced *in vitro* (ARP-4091) contributed by Dr. James E. Robinson [93]; 2G12 (ARP-1476) contributed by DAIDS/NIAID [94]; 447-52D (ARP-4030) and 257-DIV (ARP-1510) contributed by Dr. Zolla-Pazner [95,96]; 35022 (ARP-12586) contributed by Drs. Jinghe Huang and Mark Conners [97]; PG9 (ARP-11557), PG16 (ARP-12150) and PGT145 (ARP-12703) contributed by the International AIDS Vaccine Initiative [98,99]; 10–1074 (ARP-12477) contributed by Dr. Michel Nussenzweig [100]; F425-B4e8 (ARP-7626) contributed by Drs. Marshall Posner and Lisa Cavacini [101].

Anti-Human Immunodeficiency Virus Type 1 polyclonal antibodies: gp120 antiserum (sheep, ARP-288) contributed by Dr. Michael Phelan.

Entry inhibitors: Human soluble CD4-183 (sCD4-183) two-domain protein, recombinant from Escherichia coli (ARP-7356), contributed by Pharmacia, Inc; TAK-779 (ARP-4983) contributed by DAIDS/NIAID [102].

Expression vectors: HIV-1 Env_SF162_ (ARP-10463) contributed by Dr. Leonidas Stamatatos and Dr. Cecilia Cheng-Mayer [103]; HIV-1 Env_BG505_ (BG505.W6M.ENV.C2, ARP-11518) contributed by Dr. Julie Overbaugh [104]; HIV-1_NL4-3_ molecular clone (pNL4-3, ARP-2852) contributed by Dr. M. Martin [105]; HIV-1 Rev (pCMV_Rev/pRev-1, ARP-1443) contributed by Marie-Louise Hammarskjöld and Dr. David Rekosh [106].

HEK293T cells were obtained from the ATCC. Small molecule entry inhibitor AMD3100 was obtained from Sigma Aldrich. Expression vectors for HIV-1 Env_HXB2_ and Env_JRFL_ and pseudotyping vector pNL4-3 E^-^R^-^Luc^+^ were a kind gift of Dr. Benjamin Chen [107].

### Molecular biology

All *env* genes were amplified by PCR from their parental plasmids and cloned into the Xho1 and Eag1 restriction sites of the pEBB expression plasmid [107] (oligos from Eurofins, PCR enzyme Pfu Turbo from Agilent, restriction and ligation enzymes from New England Biolabs). V3-loop swaps were performed as previously described [63]. Briefly, PCR amplification of the Env_HXB2_ or Env_SF162_ V3 loop using overhang primers produced short dsDNA segments that were subsequently used as primers in QuikChange PCR (Agilent) to introduce the desired changes. Point mutations in the Env sequences were achieved through QuikChange PCR using overlapping oligonucleotides (Eurofins and IDT). All Env constructs were confirmed through Sanger sequencing of the entire open-reading frame prior to use (Genewiz).

### Peptide preparation

Preparation of C-peptide C37-KYI was performed as previously described [65]. Briefly, HIV-1 gp41 TOH construct NC1 containing N637K and T639I substitutions was expressed and purified from bacterial lysates using metal-chelate chromatography (Qiagen). Eluate was proteolyzed with trypsin (Sigma-Aldrich), and C37-KYI was purified to homogeneity by reverse phase HPLC using a Vydac C18 column and linear gradient of acetonitrile in water containing 0.1% trifluoroacetic acid. C37-KYI was lyophilized for storage and resuspended in water for use. The identity of the peptide was confirmed by mass spectrometry.

The 5-Helix protein was solubilized from bacterial inclusion bodies using 8M guanidine HCl in Tris-buffered saline (TBS) [65]. 5-Helix was recovered using metal-chelate chromatography and renatured in 4M guanidine HCl by a reverse thermal gradient (90°C to room temperature over 4hrs). Properly folded monomeric species were separated from protein aggregates by size exclusion chromatography (Superdex 75, GE Healthcare). The identity of the protein was confirmed by SDS-PAGE (molecular weight 25 kDa) and HIV-1 inhibition (activity) assays.

The expression plasmid for CD4-antagonist D23.2 (kindly provided by Dr. Alexandra Trkola, University of Zürich) was transformed into XL1 Blue cells. A 200mL starter culture of XL1 cells in LB supplemented with 1% (w/v) D-Glucose and 100 μg/ml carbenicillin (Fisher) was grown overnight and used to inoculate 1–2 L of LB plus 1% (w/v) D-Glucose and 100 μg/ml carbenicillin. When the culture achieved an OD_600_ = 0.9–1.1, expression of the DARPin was induced with 1 mM IPTG (final concentration) and the culture was left to grow for 4 hours at 37°C. Bacteria were isolated by centrifugation and resuspended in PBS500 buffer [1xPBS solution (Fisher) with an additional 500mM NaCl and 20 mM Imidazole, pH 7.2] supplemented with 100μg/mL DNaseI, 2mM PMSF and 2 μg/mL each of Leupeptin and Pepstatin (all chemicals from Sigma-Aldrich). Bacteria were lysed using a French press (500psi) and the lysate was diluted 1:2 with PBS500 containing 8 M urea. The solution was centrifuged (Sorvall SS-34) at 17,000 x g for 20 minutes at 4°C. Supernatant was applied to Ni-NTA agarose (Qiagen), and protein was renatured by reverse urea gradient over 20 column volumes and ultimately eluted in PBS500 containing 250 mM imidazole. Eluate was concentrated and subjected to size exclusion chromatography (Superdex 75) in 1xPBS to separate monomers from aggregates. D23.2 was concentrated using a 9 kDa MWCO Amicon concentrator to greater than 100 μM, flash frozen in liquid nitrogen and stored at -80°C until use. The identity of the protein was confirmed by SDS-PAGE (molecular weight 14.7 kDa) and HIV-1 inhibition (activity) assays. The concentrations of D23.2, 5-Helix and C37-KYI were determined at 280nm using the method of Edelhoch [108].

### Viral infection neutralization assays

HIV-1 infectivity assays were performed as previously described [63,64]. HIV-1 virions pseudotyped with Env variants were generated by transfecting HEK293T cells (5 x 10^6^) with Env-deficient, luciferase-expressing viral vector pNL4-3 E^-^R^-^Luc^+^ (2.5 μg) and Env-expression plasmid pEBB_Env_X_ (2.5 μg) using linear polyethylenimine (PEI, MW 25 kDa, 60 μg/ml final concentration, PolySciences). Virus-containing culture supernatant was collected 48hrs post transfection, separated from cellular debris via centrifugation (500 x g, 10min, 4°C) and stored at -80°C until use. For viral infection, target U87 cells (U87.CD4.CXCR4 or U87.CD4.CCR5) were seeded at 20,000 cells/well (96-well plate, Grenier Bio) 18–24 hours prior to viral inoculation. At the time of infection, culture supernatants were replaced with 50 μl of media with and without antibodies/inhibitors and 50 μl of virus sample. Cultures were incubated at 37°C for 48 hours before supernatant was removed and cells lysed in 1% Triton X-100. Viral infectivity was quantified by luminescence to assess expression of the luciferase reporter (luciferin from RPI or Promega; BMG Labtech Fluostar Optima plate reader).

Neutralization potencies were determined by serial dilution of antibodies and inhibitors. Each condition was tested in duplicate or triplicate in each experiment. Averaged data were normalized to the infection level measured in the absence of inhibitors and fit to the Langmuir equation ($Fraction\;Infection=\frac{1}{1+\frac{\left[Inhib\right]}{IC50}}$) unless otherwise specified. For experiments exploring the CoRA-dependence to inhibitory potency, titrations of antibody and FIs were prepared in solutions containing CoRA at defined concentrations. Experiments were repeated three or more times using at least two different production batches of the same virus (unless otherwise indicated).

For the HIV-1 preincubation/dilution experiments in S2 Fig, virus was suspended in media with and without NAb 10E8v4 at 2-4x IC50 concentration to form full-strength samples. A portion of these samples (± antibody) was diluted 10-fold either immediately or after a 1-hour incubation at 37°C. The sample diluted at 1 hour was further incubated at 37°C for 3 hours to allow for antibody unbinding and recovery from neutralization. Culture media over target cells was completely replaced with full strength or diluted samples after 0, 1 and 4 hour incubations and viral infectivity was measured by luminescence 48 hours later.

### sCD4-induced gp120 shedding assay

HEK293T cells (5 x 10^6^) were transfected with 2.5 μg each of pEBB_Env_X_ and pCMV_Rev using the PEI method described above and incubated for 48 hours. Cells were lifted with EDTA (0.5 mM in 1xPBS) and extensively washed with 1xPBS before being resuspended in 350 μL of 1xPBS containing 2 mg/ml BSA (Sigma-Aldrich), 1 μM PMSF (Sigma-Aldrich), and Complete protease inhibitor (Roche). Aliquots (50 μL) of the cell suspensions were supplemented with sCD4 (50 μL) at various concentrations and incubated for 1 hour at 37°C. Cells were pelleted by gentle centrifugation (10 minutes at 300 x g), and the clarified supernatant was subjected to SDS-PAGE (10% acrylamide, BioRad). Separated proteins were transferred to nitrocellulose paper (Amersham, GE) and probed with sheep anti-gp120 antiserum followed by HRP-conjugated donkey anti-sheep antibody (Jackson Immuno). Protein bands were visualized using ECL detection reagent (Thermo Fisher), imaged using a ChemiDoc system (BioRad), and quantified by densitometry using Adobe Photoshop. Band intensities of shed gp120 were normalized to the intensity of gp120 in a lysate (100 μL of assay buffer supplemented with 2% Triton X-100) of an equal aliquot of untreated cells.

### Cold inactivation

HIV-1 samples were prepared and harvested as described above, and aliquots (200 μL) were immediately flash frozen in liquid nitrogen before being stored at -80°C. At designated timepoints between 96 hours and 1 hour prior to infection, viral aliquots were removed from the -80°C storage, quickly thawed at 37°C for 1 min, and transferred to ice until being applied to target cells. At time of infection, aliquots on ice were collectively warmed to 37°C for 1 min before addition to U87 cells. Viral infection was measured 48 hours later as described above. For each experiment, data were normalized to the infection level of the 1-hour timepoint.

## Supporting information

S1 Table IC50 values (μg/ml) for different NAbs grouped by tier designation. Numbers reflect the geometric means obtained from viruses with both detected and undetected IC50 values. Orange designates geometric means calculated from groups in which less than 85% of the IC50 values were measurable. Data were procured from the Los Alamos National Laboratory HIV Immunology database (January 2022). For comparison, the mean IC50 values for H4, H5 and H4 R306S Envs found in this study are shown. Red numbers indicate that 50% neutralization was not achieved at the highest concentrations tested.
(DOCX)

S2 Table IC50 values for different NAbs and sCD4 against Envs with V3-loop swaps and residue 306 substitutions. Numbers reflect the mean and SEM from three or more independent experiments. The units are ng/ml for all NAbs and nM for sCD4. Red numbers indicate that 50% neutralization was not achieved at the highest concentrations tested. Blank spaces indicate that the antibody or sCD4 was not tested against that Env. Note that B4 R306S Env was nonfunctional.
(DOCX)

S1 Fig Relative infectivity of HIV-1 pseudotyped with different wild type and variant Env species. **(A)** For each virus, infectivity was measured on both U87.CD4.CXCR4 cells (grey bars) and U87.CD4.CCR5 (magenta bars) and normalized to viral content estimated by luciferase expression from proviral DNA in virus-producing cells (see **B**). Normalized infectivity is reported relative to the values for H4 Env on CXCR4^+^ cells and H5 Env on CCR5^+^ cells. Data represent the mean±range-of-mean from two independent experiments. **(B)** Relationship between luciferase expression from virus producing cells and HIV-1 p24 levels found in culture supernatants. For each experiment, cells were transfected (Lipofectamine Plus, Life Sciences) with differing amounts of pseudotyping vector pNL4-3 E^-^R^-^Luc^+^, Env-expressing vector pEBB_HXB2Env and GFP-expressing vector pBABE_GFP as indicated in the legend. After a 36 hour incubation, supernatants were collected and assayed for viral p24 content by ELISA (see reference [63] of text) while cells were lysed and assessed for luciferase activity. For each experiment, the measured solution p24 levels and luciferase activities were normalized to their respective values obtained from the 0.5 μg pNL4-3 E^-^R^-^Luc^+^ transfection. Data from four separate experiments have been fit to a linear regression fixed to go through the origin; the slope was 0.96 and the R-value was 0.93.
(TIF)

S2 Fig V3-loop changes in Env_HXB2_ do not alter the irreversibility of anti-MPER NAb 10E8v4 neutralization. (**A**) Experimental timeline of the antibody dilution experiment. HIV-1 was suspended in media with and without NAb 10E8v4 at 2-4x IC50 concentration to form the full-strength samples. A portion of these samples (± antibody) was diluted 10-fold either immediately or after a 1-hour incubation at 37°C. The sample diluted at 1 hour was further incubated at 37°C for 3 hours to allow for antibody unbinding and recovery from neutralization. Culture media over U87 target cells was completely replaced with full strength or diluted samples at the 0-, 1- and 4- hour time points (red circles) to asses viral infectivity in the presence or absence of antibody. (**B**) Representative data from a single experiment using HIV-1 pseudotyped with H4 Env. Infectivity was measured by luciferase reporter expression for full strength (FS) or diluted (Dil) samples at the indicated time points. Bars and error bars represent the mean ± SEM of three experimental replicates for each sample. (**C-F**) NAb 10E8v4 neutralization of HIV-1 pseudotyped with H4 (**C**), H4 R306S (**D**), H5 (**E**) and J5 (**F**) Envs. Fraction infectivity was calculated as the ratio of the luciferase signals recorded in the presence and absence of NAb 10E8v4 (i.e., adjacent magenta and cyan bars in **B**) under each condition (FS or Dil) at the indicated time point. Antibody concentrations (ng/mL) in the FS and Dil conditions, as well as the IC50 value, are listed in the adjacent box. Data represent the mean ± SEM from four to five independent experiments. The lower fraction infectivity at the 1-hour timepoint compared to the 0-hour timepoint (more easily seen in the Dil samples) indicated that antibody binding to the prefusogenic state occurred during the 0-to-1 hour incubation period under FS conditions. Neutralization was enhanced by approximately the same amount for all Envs tested despite the fact that NAb 10E8 concentrations in H4 R306S, H5 and J5 Env viral samples were approximately 10- to 20-fold higher than that in wild type H4 Env samples. This finding suggested that the antibody association rate constant was much greater for H4 Env than for the other Env species. The lower fraction infectivity in the Dil samples at the 4-hour timepoint compared to the 1-hour timepoint implied that antibody dissociation was slow compared to 10E8-induced Env inactivation (see reference [22] of main text). The functional irreversibility of NAb 10E8 neutralization ruled out decreased binding strength (manifest in increased dissociation rate) as the mechanism behind the reduced antibody potency caused by the V3-loop swap or R306S substitution in Env_HXB2_. Together, the results implied that disruption of anti-MPER antibody neutralization arose from a reduced association rate constant, consistent with a decreased lifetime of an MPER-accessible Env conformation.
(TIF)

S3 Fig Heat inactivation of HIV-1 pseudotyped with H4, H5 or J5 Env. Viral aliquots were incubated at various temperatures for 45 minutes prior to addition to U87 cell cultures at 37°C. Infectivity levels were normalized to the level measured following a 37°C incubation. Data represent the mean±range-of-mean from two independent experiments.
(TIF)

S4 FigDependence of viral infectivity on cellular CD4 and CoR levels.**(A-E)** Infectivity levels of pseudotyped HIV-1 were measured in the presence of CD4 antagonist (D23.2) or CoRA (AMD3100 or TAK-779) alone or in combination. U87.CD4.CXCR4 target cells were used for H4 Env (**A**) and Env_89.6_ (**D**), while U87.CD4.CCR5 target cells were used for H5 Env (**B**), J5 Env (**C**) and Env_89.6_ (**E**). Data were normalized to the level obtained in the absence of both inhibitors. (**F-J**) The same data in **A-E** except plotted as D23.2 titrations for each CoRA concentration. Data represent the mean±SEM from seven independent experiments and have been fit to the Hill Equation:

$$Fraction\;Infection=\frac{I_{0}}{1+{\left(\frac{\left[D32.2\right]}{IC50}\right)}^{n_{H}}}$$

where I_0_ represents the normalized infection level in the presence of CoRA but the absence of D23.2 and n_H_ is the Hill coefficient. Data were reliably fit for relative infection levels greater than 0.005. (**K-O**) Relative infection level (I_o_), IC50 and Hill coefficient (n_H_) values extracted from the data fits in **F-J**.(TIF)

S5 Fig Sensitivity of J4 and J5 Envs to gp41-targeted inhibitors. (**A-C**) Inhibitory titrations of C37-KYI (**A**), 5-Helix (**B**) and 10E8 (**C**) against HIV-1 pseudotyped with J4 (open) and J5 (closed) Envs. (**D**) Table of IC50 values. All data reflect the mean±SEM of four or more independent experiments. For all three inhibitors, J4 Env had significantly lower IC50 values than J5 Env (two-sample t-test with equal variance not assumed, p-value in table).
(TIF)

S6 Fig Evidence for stabilization of the V1/V2- and V3-loop interaction through residue coupling spatially adjacent to residue 306. (**A**) Ribbon diagram of the V1/V2 and V3 loops in Env_JRFL_ SOSIP.664 (PDB 5FYK, reference [79] of main text) highlighting residues at position 306 (Ser-black) and 318 (Tyr-cyan). The same amino acids are found at positions 306 and 318 in H5 Env; in H4 Env, the amino acids are Arg306 and Val318. **(B)** Table of sequence conservation at V1/V2-loop residues 172 and 173 and V3-loop residues 305, 306 and 318 grouped by HIV-1 clade. Numbers represent summed frequencies of the indicated amino acids. (**C**) NAb 10E8v4 sensitivity of H4, H4 R306S and H5 Envs with the indicated substitutions at residue 318. Data from the Env variants with mutations at residue 318 represent the mean±range-of-mean from two independent experiments. Data from other variants represent mean±SEM of more than three independent experiments. **(D)** Frequency of a potential ionic interaction between V1/V2-loop residue 172 and V3-loop residue 305 grouped by clade. (**E**) Ribbon diagram of the V1/V2 and V3 loops in Env_BG505_ SOSIP.664 structure (PDB 4ZMJ, reference [83] of main text) highlighting residues at position 173 (Tyr-blue), 305 (Lys-green) and 306 (Ser-black). **(F)** Frequency of a potential cation-pi interaction between V1/V2-loop residue 173 and V3-loop residue 305 grouped by clade. Sequence data obtained from the Los Alamos National Laboratory HIV Sequence database (October 2021).
(TIF)

## References

[1] Xiao T, Cai Y, Chen B. HIV-1 Entry and Membrane Fusion Inhibitors. Viruses. 2021;13(5). Epub 20210423. doi: 10.3390/v13050735 ; PubMed Central PMCID: PMC8146413. 33922579 PMC8146413

[2] Pancera M, Changela A, Kwong PD. How HIV-1 entry mechanism and broadly neutralizing antibodies guide structure-based vaccine design. Curr Opin HIV AIDS. 2017;12(3):229–40. Epub 2017/04/20. doi: 10.1097/COH.0000000000000360 ; PubMed Central PMCID: PMC5557343. 28422787 PMC5557343

[3] Ward AB, Wilson IA. The HIV-1 envelope glycoprotein structure: nailing down a moving target. Immunol Rev. 2017;275(1):21–32. Epub 2017/01/31. doi: 10.1111/imr.12507 ; PubMed Central PMCID: PMC5300090. 28133813 PMC5300090

[4] Ng’uni T, Chasara C, Ndhlovu ZM. Major Scientific Hurdles in HIV Vaccine Development: Historical Perspective and Future Directions. Front Immunol. 2020;11:590780. Epub 20201028. doi: 10.3389/fimmu.2020.590780 ; PubMed Central PMCID: PMC7655734. 33193428 PMC7655734

[5] Klasse PJ, Ozorowski G, Sanders RW, Moore JP. Env Exceptionalism: Why Are HIV-1 Env Glycoproteins Atypical Immunogens? Cell Host Microbe. 2020;27(4):507–18. Epub 2020/04/10. doi: 10.1016/j.chom.2020.03.018 ; PubMed Central PMCID: PMC7187920. 32272076 PMC7187920

[6] Checkley MA, Luttge BG, Freed EO. HIV-1 envelope glycoprotein biosynthesis, trafficking, and incorporation. J Mol Biol. 2011;410(4):582–608. doi: 10.1016/j.jmb.2011.04.042 ; PubMed Central PMCID: PMC3139147. 21762802 PMC3139147

[7] Bennett AL, Henderson R. HIV-1 Envelope Conformation, Allostery, and Dynamics. Viruses. 2021;13(5). Epub 20210507. doi: 10.3390/v13050852 ; PubMed Central PMCID: PMC8150877. 34067073 PMC8150877

[8] Lyumkis D, Julien JP, de Val N, Cupo A, Potter CS, Klasse PJ, et al. Cryo-EM structure of a fully glycosylated soluble cleaved HIV-1 envelope trimer. Science. 2013;342(6165):1484–90. Epub 20131031. doi: 10.1126/science.1245627 ; PubMed Central PMCID: PMC3954647. 24179160 PMC3954647

[9] Pancera M, Zhou T, Druz A, Georgiev IS, Soto C, Gorman J, et al. Structure and immune recognition of trimeric pre-fusion HIV-1 Env. Nature. 2014;514(7523):455–61. Epub 20141008. doi: 10.1038/nature13808 ; PubMed Central PMCID: PMC4348022. 25296255 PMC4348022

[10] Tran EE, Borgnia MJ, Kuybeda O, Schauder DM, Bartesaghi A, Frank GA, et al. Structural mechanism of trimeric HIV-1 envelope glycoprotein activation. PLoS Pathog. 2012;8(7):e1002797. Epub 20120712. doi: 10.1371/journal.ppat.1002797 ; PubMed Central PMCID: PMC3395603. 22807678 PMC3395603

[11] Wang H, Barnes CO, Yang Z, Nussenzweig MC, Bjorkman PJ. Partially Open HIV-1 Envelope Structures Exhibit Conformational Changes Relevant for Coreceptor Binding and Fusion. Cell Host Microbe. 2018;24(4):579–92 e4. Epub 2018/10/12. doi: 10.1016/j.chom.2018.09.003 ; PubMed Central PMCID: PMC6185872. 30308160 PMC6185872

[12] Ozorowski G, Pallesen J, de Val N, Lyumkis D, Cottrell CA, Torres JL, et al. Open and closed structures reveal allostery and pliability in the HIV-1 envelope spike. Nature. 2017;547(7663):360–3. Epub 20170712. doi: 10.1038/nature23010 ; PubMed Central PMCID: PMC5538736. 28700571 PMC5538736

[13] Wang H, Cohen AA, Galimidi RP, Gristick HB, Jensen GJ, Bjorkman PJ. Cryo-EM structure of a CD4-bound open HIV-1 envelope trimer reveals structural rearrangements of the gp120 V1V2 loop. Proc Natl Acad Sci U S A. 2016;113(46):E7151–E8. Epub 20161031. doi: 10.1073/pnas.1615939113 ; PubMed Central PMCID: PMC5135367. 27799557 PMC5135367

[14] Li Z, Li W, Lu M, Bess J Jr., Chao CW, Gorman J, et al. Subnanometer structures of HIV-1 envelope trimers on aldrithiol-2-inactivated virus particles. Nat Struct Mol Biol. 2020;27(8):726–34. Epub 20200629. doi: 10.1038/s41594-020-0452-2 ; PubMed Central PMCID: PMC8138683. 32601441 PMC8138683

[15] Sattentau QJ, Moore JP. Conformational changes induced in the human immunodeficiency virus envelope glycoprotein by soluble CD4 binding. J Exp Med. 1991;174(2):407–15. doi: 10.1084/jem.174.2.407 ; PubMed Central PMCID: PMC2118908. 1713252 PMC2118908

[16] Kwong PD, Wyatt R, Robinson J, Sweet RW, Sodroski J, Hendrickson WA. Structure of an HIV gp120 envelope glycoprotein in complex with the CD4 receptor and a neutralizing human antibody. Nature. 1998;393(6686):648–59. doi: 10.1038/31405 ; PubMed Central PMCID: PMC5629912. 9641677 PMC5629912

[17] Sattentau QJ, Zolla-Pazner S, Poignard P. Epitope exposure on functional, oligomeric HIV-1 gp41 molecules. Virology. 1995;206(1):713–7. doi: 10.1016/s0042-6822(95)80094-8 . 7530400

[18] Shaik MM, Peng H, Lu J, Rits-Volloch S, Xu C, Liao M, et al. Structural basis of coreceptor recognition by HIV-1 envelope spike. Nature. 2019;565(7739):318–23. Epub 20181212. doi: 10.1038/s41586-018-0804-9 ; PubMed Central PMCID: PMC6391877. 30542158 PMC6391877

[19] Berger EA, Murphy PM, Farber JM. Chemokine receptors as HIV-1 coreceptors: roles in viral entry, tropism, and disease. Annu Rev Immunol. 1999;17:657–700. doi: 10.1146/annurev.immunol.17.1.657 . 10358771

[20] Melikyan GB, Platt EJ, Kabat D. The role of the N-terminal segment of CCR5 in HIV-1 Env-mediated membrane fusion and the mechanism of virus adaptation to CCR5 lacking this segment. Retrovirology. 2007;4:55. Epub 20070808. doi: 10.1186/1742-4690-4-55 ; PubMed Central PMCID: PMC1995219. 17686153 PMC1995219

[21] Moore JP, McKeating JA, Weiss RA, Sattentau QJ. Dissociation of gp120 from HIV-1 virions induced by soluble CD4. Science. 1990;250(4984):1139–42. doi: 10.1126/science.2251501 2251501

[22] Ruprecht CR, Krarup A, Reynell L, Mann AM, Brandenberg OF, Berlinger L, et al. MPER-specific antibodies induce gp120 shedding and irreversibly neutralize HIV-1. J Exp Med. 2011;208(3):439–54. Epub 20110228. doi: 10.1084/jem.20101907 ; PubMed Central PMCID: PMC3058584. 21357743 PMC3058584

[23] Kliger Y, Aharoni A, Rapaport D, Jones P, Blumenthal R, Shai Y. Fusion peptides derived from the HIV type 1 glycoprotein 41 associate within phospholipid membranes and inhibit cell-cell Fusion. Structure-function study. J Biol Chem. 1997;272(21):13496–505. doi: 10.1074/jbc.272.21.13496 . 9153194

[24] Dimitrov AS, Xiao X, Dimitrov DS, Blumenthal R. Early intermediates in HIV-1 envelope glycoprotein-mediated fusion triggered by CD4 and co-receptor complexes. J Biol Chem. 2001;276(32):30335–41. Epub 20010607. doi: 10.1074/jbc.M103788200 . 11397808

[25] Cai L, Gochin M, Liu K. Biochemistry and biophysics of HIV-1 gp41—membrane interactions and implications for HIV-1 envelope protein mediated viral-cell fusion and fusion inhibitor design. Curr Top Med Chem. 2011;11(24):2959–84. Epub 2011/11/03. doi: 10.2174/156802611798808497 ; PubMed Central PMCID: PMC3220743. 22044229 PMC3220743

[26] Weissenhorn W, Dessen A, Harrison SC, Skehel JJ, Wiley DC. Atomic structure of the ectodomain from HIV-1 gp41. Nature. 1997;387(6631):426–30. doi: 10.1038/387426a0 . 9163431

[27] Chan DC, Fass D, Berger JM, Kim PS. Core structure of gp41 from the HIV envelope glycoprotein. Cell. 1997;89(2):263–73. doi: 10.1016/s0092-8674(00)80205-6 . 9108481

[28] Buzon V, Natrajan G, Schibli D, Campelo F, Kozlov MM, Weissenhorn W. Crystal structure of HIV-1 gp41 including both fusion peptide and membrane proximal external regions. PLoS Pathog. 2010;6(5):e1000880. Epub 20100506. doi: 10.1371/journal.ppat.1000880 ; PubMed Central PMCID: PMC2865522. 20463810 PMC2865522

[29] Caillat C, Guilligay D, Torralba J, Friedrich N, Nieva JL, Trkola A, et al. Structure of HIV-1 gp41 with its membrane anchors targeted by neutralizing antibodies. Elife. 2021;10. Epub 20210419. doi: 10.7554/eLife.65005 ; PubMed Central PMCID: PMC8084527. 33871352 PMC8084527

[30] Harrison SC. Viral membrane fusion. Nat Struct Mol Biol. 2008;15(7):690–8. doi: 10.1038/nsmb.1456 ; PubMed Central PMCID: PMC2517140. 18596815 PMC2517140

[31] Weissenhorn W, Hinz A, Gaudin Y. Virus membrane fusion. FEBS Lett. 2007;581(11):2150–5. Epub 20070216. doi: 10.1016/j.febslet.2007.01.093 ; PubMed Central PMCID: PMC7094569. 17320081 PMC7094569

[32] Munro JB, Mothes W. Structure and Dynamics of the Native HIV-1 Env Trimer. J Virol. 2015;89(11):5752–5. Epub 20150311. doi: 10.1128/JVI.03187-14 ; PubMed Central PMCID: PMC4442439. 25762739 PMC4442439

[33] Stadtmueller BM, Bridges MD, Dam KM, Lerch MT, Huey-Tubman KE, Hubbell WL, et al. DEER Spectroscopy Measurements Reveal Multiple Conformations of HIV-1 SOSIP Envelopes that Show Similarities with Envelopes on Native Virions. Immunity. 2018;49(2):235–46 e4. Epub 20180731. doi: 10.1016/j.immuni.2018.06.017 ; PubMed Central PMCID: PMC6104740. 30076100 PMC6104740

[34] Henderson R, Lu M, Zhou Y, Mu Z, Parks R, Han Q, et al. Disruption of the HIV-1 Envelope allosteric network blocks CD4-induced rearrangements. Nat Commun. 2020;11(1):520. Epub 20200124. doi: 10.1038/s41467-019-14196-w ; PubMed Central PMCID: PMC6981184. 31980614 PMC6981184

[35] Munro JB, Gorman J, Ma X, Zhou Z, Arthos J, Burton DR, et al. Conformational dynamics of single HIV-1 envelope trimers on the surface of native virions. Science. 2014;346(6210):759–63. Epub 20141008. doi: 10.1126/science.1254426 ; PubMed Central PMCID: PMC4304640. 25298114 PMC4304640

[36] Ma X, Lu M, Gorman J, Terry DS, Hong X, Zhou Z, et al. HIV-1 Env trimer opens through an asymmetric intermediate in which individual protomers adopt distinct conformations. Elife. 2018;7. Epub 20180321. doi: 10.7554/eLife.34271 ; PubMed Central PMCID: PMC5896952. 29561264 PMC5896952

[37] Lu M, Ma X, Castillo-Menendez LR, Gorman J, Alsahafi N, Ermel U, et al. Associating HIV-1 envelope glycoprotein structures with states on the virus observed by smFRET. Nature. 2019;568(7752):415–9. Epub 20190410. doi: 10.1038/s41586-019-1101-y ; PubMed Central PMCID: PMC6655592. 30971821 PMC6655592

[38] Boliar S, Patil S, Shukla BN, Ghobbeh A, Deshpande S, Chen W, et al. Ligand accessibility to the HIV-1 Env co-receptor binding site can occur prior to CD4 engagement and is independent of viral tier category. Virology. 2018;519:99–105. Epub 20180511. doi: 10.1016/j.virol.2018.04.002 . 29684630

[39] Seaman MS, Janes H, Hawkins N, Grandpre LE, Devoy C, Giri A, et al. Tiered categorization of a diverse panel of HIV-1 Env pseudoviruses for assessment of neutralizing antibodies. J Virol. 2010;84(3):1439–52. Epub 20091125. doi: 10.1128/JVI.02108-09 ; PubMed Central PMCID: PMC2812321. 19939925 PMC2812321

[40] Ivan B, Sun Z, Subbaraman H, Friedrich N, Trkola A. CD4 occupancy triggers sequential pre-fusion conformational states of the HIV-1 envelope trimer with relevance for broadly neutralizing antibody activity. PLoS Biol. 2019;17(1):e3000114. Epub 20190116. doi: 10.1371/journal.pbio.3000114 ; PubMed Central PMCID: PMC6351000. 30650070 PMC6351000

[41] Guttman M, Cupo A, Julien JP, Sanders RW, Wilson IA, Moore JP, et al. Antibody potency relates to the ability to recognize the closed, pre-fusion form of HIV Env. Nat Commun. 2015;6:6144. Epub 20150205. doi: 10.1038/ncomms7144 ; PubMed Central PMCID: PMC4338595. 25652336 PMC4338595

[42] Flemming J, Wiesen L, Herschhorn A. Conformation-Dependent Interactions Between HIV-1 Envelope Glycoproteins and Broadly Neutralizing Antibodies. AIDS Res Hum Retroviruses. 2018;34(9):794–803. Epub 20180717. doi: 10.1089/AID.2018.0102 . 29905080

[43] Haim H, Strack B, Kassa A, Madani N, Wang L, Courter JR, et al. Contribution of intrinsic reactivity of the HIV-1 envelope glycoproteins to CD4-independent infection and global inhibitor sensitivity. PLoS Pathog. 2011;7(6):e1002101. Epub 20110623. doi: 10.1371/journal.ppat.1002101 ; PubMed Central PMCID: PMC3121797. 21731494 PMC3121797

[44] McGee K, Haim H, Korioth-Schmitz B, Espy N, Javanbakht H, Letvin N, et al. The selection of low envelope glycoprotein reactivity to soluble CD4 and cold during simian-human immunodeficiency virus infection of rhesus macaques. J Virol. 2014;88(1):21–40. Epub 20131016. doi: 10.1128/JVI.01558-13 ; PubMed Central PMCID: PMC3911730. 24131720 PMC3911730

[45] Johnston SH, Lobritz MA, Nguyen S, Lassen K, Delair S, Posta F, et al. A quantitative affinity-profiling system that reveals distinct CD4/CCR5 usage patterns among human immunodeficiency virus type 1 and simian immunodeficiency virus strains. J Virol. 2009;83(21):11016–26. Epub 20090819. doi: 10.1128/JVI.01242-09 ; PubMed Central PMCID: PMC2772777. 19692480 PMC2772777

[46] Chikere K, Chou T, Gorry PR, Lee B. Affinofile profiling: how efficiency of CD4/CCR5 usage impacts the biological and pathogenic phenotype of HIV. Virology. 2013;435(1):81–91. doi: 10.1016/j.virol.2012.09.043 ; PubMed Central PMCID: PMC3522187. 23217618 PMC3522187

[47] Lu M, Ma X, Reichard N, Terry DS, Arthos J, Smith AB, 3rd, et al. Shedding-Resistant HIV-1 Envelope Glycoproteins Adopt Downstream Conformations That Remain Responsive to Conformation-Preferring Ligands. J Virol. 2020;94(17). Epub 20200817. doi: 10.1128/JVI.00597-20 ; PubMed Central PMCID: PMC7431789. 32522853 PMC7431789

[48] Chakrabarti BK, Walker LM, Guenaga JF, Ghobbeh A, Poignard P, Burton DR, et al. Direct antibody access to the HIV-1 membrane-proximal external region positively correlates with neutralization sensitivity. J Virol. 2011;85(16):8217–26. Epub 20110608. doi: 10.1128/JVI.00756-11 ; PubMed Central PMCID: PMC3147955. 21653673 PMC3147955

[49] Herschhorn A, Ma X, Gu C, Ventura JD, Castillo-Menendez L, Melillo B, et al. Release of gp120 Restraints Leads to an Entry-Competent Intermediate State of the HIV-1 Envelope Glycoproteins. mBio. 2016;7(5). Epub 20161025. doi: 10.1128/mBio.01598-16 ; PubMed Central PMCID: PMC5080382. 27795397 PMC5080382

[50] Medjahed H, Pacheco B, Desormeaux A, Sodroski J, Finzi A. The HIV-1 gp120 major variable regions modulate cold inactivation. J Virol. 2013;87(7):4103–11. Epub 20130123. doi: 10.1128/JVI.03124-12 ; PubMed Central PMCID: PMC3624226. 23345516 PMC3624226

[51] Herschhorn A, Gu C, Moraca F, Ma X, Farrell M, Smith AB, 3rd, et al. The beta20-beta21 of gp120 is a regulatory switch for HIV-1 Env conformational transitions. Nat Commun. 2017;8(1):1049. Epub 20171019. doi: 10.1038/s41467-017-01119-w ; PubMed Central PMCID: PMC5648922. 29051495 PMC5648922

[52] Desormeaux A, Coutu M, Medjahed H, Pacheco B, Herschhorn A, Gu C, et al. The highly conserved layer-3 component of the HIV-1 gp120 inner domain is critical for CD4-required conformational transitions. J Virol. 2013;87(5):2549–62. Epub 20121219. doi: 10.1128/JVI.03104-12 ; PubMed Central PMCID: PMC3571356. 23255784 PMC3571356

[53] Powell RLR, Totrov M, Itri V, Liu X, Fox A, Zolla-Pazner S. Plasticity and Epitope Exposure of the HIV-1 Envelope Trimer. J Virol. 2017;91(17). Epub 20170810. doi: 10.1128/JVI.00410-17 ; PubMed Central PMCID: PMC5553165. 28615206 PMC5553165

[54] Bradley T, Trama A, Tumba N, Gray E, Lu X, Madani N, et al. Amino Acid Changes in the HIV-1 gp41 Membrane Proximal Region Control Virus Neutralization Sensitivity. EBioMedicine. 2016;12:196–207. Epub 20160831. doi: 10.1016/j.ebiom.2016.08.045 ; PubMed Central PMCID: PMC5078591. 27612593 PMC5078591

[55] Leaman DP, Zwick MB. Increased functional stability and homogeneity of viral envelope spikes through directed evolution. PLoS Pathog. 2013;9(2):e1003184. Epub 20130228. doi: 10.1371/journal.ppat.1003184 ; PubMed Central PMCID: PMC3585149. 23468626 PMC3585149

[56] Gift SK, Leaman DP, Zhang L, Kim AS, Zwick MB. Functional Stability of HIV-1 Envelope Trimer Affects Accessibility to Broadly Neutralizing Antibodies at Its Apex. J Virol. 2017;91(24). Epub 20171130. doi: 10.1128/JVI.01216-17 ; PubMed Central PMCID: PMC5709597. 28978711 PMC5709597

[57] Zhang P, Kwon AL, Guzzo C, Liu Q, Schmeisser H, Miao H, et al. Functional Anatomy of the Trimer Apex Reveals Key Hydrophobic Constraints That Maintain the HIV-1 Envelope Spike in a Closed State. mBio. 2021;12(2). Epub 20210330. doi: 10.1128/mBio.00090-21 ; PubMed Central PMCID: PMC8092198. 33785631 PMC8092198

[58] Zhu CB, Zhu L, Holz-Smith S, Matthews TJ, Chen CH. The role of the third beta strand in gp120 conformation and neutralization sensitivity of the HIV-1 primary isolate DH012. Proc Natl Acad Sci U S A. 2001;98(26):15227–32. Epub 20011204. doi: 10.1073/pnas.261359098 ; PubMed Central PMCID: PMC65011. 11734627 PMC65011

[59] Musich T, Peters PJ, Duenas-Decamp MJ, Gonzalez-Perez MP, Robinson J, Zolla-Pazner S, et al. A conserved determinant in the V1 loop of HIV-1 modulates the V3 loop to prime low CD4 use and macrophage infection. J Virol. 2011;85(5):2397–405. Epub 20101215. doi: 10.1128/JVI.02187-10 ; PubMed Central PMCID: PMC3067776. 21159865 PMC3067776

[60] Zolla-Pazner S, Cohen SS, Boyd D, Kong XP, Seaman M, Nussenzweig M, et al. Structure/Function Studies Involving the V3 Region of the HIV-1 Envelope Delineate Multiple Factors That Affect Neutralization Sensitivity. J Virol. 2016;90(2):636–49. Epub 20151021. doi: 10.1128/JVI.01645-15 ; PubMed Central PMCID: PMC4702699. 26491157 PMC4702699

[61] Guzzo C, Zhang P, Liu Q, Kwon AL, Uddin F, Wells AI, et al. Structural Constraints at the Trimer Apex Stabilize the HIV-1 Envelope in a Closed, Antibody-Protected Conformation. mBio. 2018;9(6). Epub 20181211. doi: 10.1128/mBio.00955-18 ; PubMed Central PMCID: PMC6299476. 30538178 PMC6299476

[62] Mishra N, Sharma S, Dobhal A, Kumar S, Chawla H, Singh R, et al. A Rare Mutation in an Infant-Derived HIV-1 Envelope Glycoprotein Alters Interprotomer Stability and Susceptibility to Broadly Neutralizing Antibodies Targeting the Trimer Apex. J Virol. 2020;94(19). Epub 20200915. doi: 10.1128/JVI.00814-20 ; PubMed Central PMCID: PMC7495384. 32669335 PMC7495384

[63] Khasnis MD, Halkidis K, Bhardwaj A, Root MJ. Receptor Activation of HIV-1 Env Leads to Asymmetric Exposure of the gp41 Trimer. PLoS Pathog. 2016;12(12):e1006098. Epub 20161219. doi: 10.1371/journal.ppat.1006098 ; PubMed Central PMCID: PMC5222517. 27992602 PMC5222517

[64] Ahn KW, Root MJ. Complex interplay of kinetic factors governs the synergistic properties of HIV-1 entry inhibitors. J Biol Chem. 2017;292(40):16498–510. Epub 20170710. doi: 10.1074/jbc.M117.791731 ; PubMed Central PMCID: PMC5633110. 28696261 PMC5633110

[65] Kahle KM, Steger HK, Root MJ. Asymmetric deactivation of HIV-1 gp41 following fusion inhibitor binding. PLoS Pathog. 2009;5(11):e1000674. Epub 20091126. doi: 10.1371/journal.ppat.1000674 ; PubMed Central PMCID: PMC2776349. 19956769 PMC2776349

[66] Steger HK, Root MJ. Kinetic dependence to HIV-1 entry inhibition. J Biol Chem. 2006;281(35):25813–21. Epub 20060627. doi: 10.1074/jbc.M601457200 . 16803885

[67] Ofek G, Tang M, Sambor A, Katinger H, Mascola JR, Wyatt R, et al. Structure and mechanistic analysis of the anti-human immunodeficiency virus type 1 antibody 2F5 in complex with its gp41 epitope. J Virol. 2004;78(19):10724–37. doi: 10.1128/JVI.78.19.10724-10737.2004 ; PubMed Central PMCID: PMC516390. 15367639 PMC516390

[68] Murin CD, Julien JP, Sok D, Stanfield RL, Khayat R, Cupo A, et al. Structure of 2G12 Fab2 in complex with soluble and fully glycosylated HIV-1 Env by negative-stain single-particle electron microscopy. J Virol. 2014;88(17):10177–88. Epub 20140625. doi: 10.1128/JVI.01229-14 ; PubMed Central PMCID: PMC4136306. 24965454 PMC4136306

[69] Sanders RW, Venturi M, Schiffner L, Kalyanaraman R, Katinger H, Lloyd KO, et al. The mannose-dependent epitope for neutralizing antibody 2G12 on human immunodeficiency virus type 1 glycoprotein gp120. J Virol. 2002;76(14):7293–305. doi: 10.1128/jvi.76.14.7293-7305.2002 ; PubMed Central PMCID: PMC136300. 12072528 PMC136300

[70] Chuang GY, Zhou J, Acharya P, Rawi R, Shen CH, Sheng Z, et al. Structural Survey of Broadly Neutralizing Antibodies Targeting the HIV-1 Env Trimer Delineates Epitope Categories and Characteristics of Recognition. Structure. 2019;27(1):196–206 e6. Epub 20181121. doi: 10.1016/j.str.2018.10.007 ; PubMed Central PMCID: PMC6664815. 30471922 PMC6664815

[71] Wang H, Gristick HB, Scharf L, West AP, Galimidi RP, Seaman MS, et al. Asymmetric recognition of HIV-1 Envelope trimer by V1V2 loop-targeting antibodies. Elife. 2017;6. Epub 20170526. doi: 10.7554/eLife.27389 ; PubMed Central PMCID: PMC5472438. 28548638 PMC5472438

[72] Frey G, Peng H, Rits-Volloch S, Morelli M, Cheng Y, Chen B. A fusion-intermediate state of HIV-1 gp41 targeted by broadly neutralizing antibodies. Proc Natl Acad Sci U S A. 2008;105(10):3739–44. Epub 20080305. doi: 10.1073/pnas.0800255105 ; PubMed Central PMCID: PMC2268799. 18322015 PMC2268799

[73] Dimitrov AS, Jacobs A, Finnegan CM, Stiegler G, Katinger H, Blumenthal R. Exposure of the membrane-proximal external region of HIV-1 gp41 in the course of HIV-1 envelope glycoprotein-mediated fusion. Biochemistry. 2007;46(5):1398–401. doi: 10.1021/bi062245f . 17260969

[74] Platt EJ, Durnin JP, Kabat D. Kinetic factors control efficiencies of cell entry, efficacies of entry inhibitors, and mechanisms of adaptation of human immunodeficiency virus. J Virol. 2005;79(7):4347–56. doi: 10.1128/JVI.79.7.4347-4356.2005 ; PubMed Central PMCID: PMC1061535. 15767435 PMC1061535

[75] Agrawal N, Leaman DP, Rowcliffe E, Kinkead H, Nohria R, Akagi J, et al. Functional stability of unliganded envelope glycoprotein spikes among isolates of human immunodeficiency virus type 1 (HIV-1). PLoS One. 2011;6(6):e21339. Epub 20110627. doi: 10.1371/journal.pone.0021339 ; PubMed Central PMCID: PMC3124497. 21738637 PMC3124497

[76] Schweizer A, Rusert P, Berlinger L, Ruprecht CR, Mann A, Corthesy S, et al. CD4-specific designed ankyrin repeat proteins are novel potent HIV entry inhibitors with unique characteristics. PLoS Pathog. 2008;4(7):e1000109. Epub 20080725. doi: 10.1371/journal.ppat.1000109 ; PubMed Central PMCID: PMC2453315. 18654624 PMC2453315

[77] Pugach P, Kuhmann SE, Taylor J, Marozsan AJ, Snyder A, Ketas T, et al. The prolonged culture of human immunodeficiency virus type 1 in primary lymphocytes increases its sensitivity to neutralization by soluble CD4. Virology. 2004;321(1):8–22. Epub 2004/03/23. doi: 10.1016/j.virol.2003.12.012 . 15033560

[78] Zhou T, Georgiev I, Wu X, Yang ZY, Dai K, Finzi A, et al. Structural basis for broad and potent neutralization of HIV-1 by antibody VRC01. Science. 2010;329(5993):811–7. Epub 20100708. doi: 10.1126/science.1192819 ; PubMed Central PMCID: PMC2981354. 20616231 PMC2981354

[79] Stewart-Jones GB, Soto C, Lemmin T, Chuang GY, Druz A, Kong R, et al. Trimeric HIV-1-Env Structures Define Glycan Shields from Clades A, B, and G. Cell. 2016;165(4):813–26. Epub 20160421. doi: 10.1016/j.cell.2016.04.010 ; PubMed Central PMCID: PMC5543418. 27114034 PMC5543418

[80] Rawi R, Kunji K, Haoudi A, Bensmail H. Coevolution Analysis of HIV-1 Envelope Glycoprotein Complex. PLoS One. 2015;10(11):e0143245. Epub 20151118. doi: 10.1371/journal.pone.0143245 ; PubMed Central PMCID: PMC4651434. 26579711 PMC4651434

[81] Mangala Prasad V, Leaman DP, Lovendahl KN, Croft JT, Benhaim MA, Hodge EA, et al. Cryo-ET of Env on intact HIV virions reveals structural variation and positioning on the Gag lattice. Cell. 2022;185(4):641–53 e17. Epub 20220204. doi: 10.1016/j.cell.2022.01.013 . 35123651 PMC9000915

[82] Rantalainen K, Berndsen ZT, Antanasijevic A, Schiffner T, Zhang X, Lee WH, et al. HIV-1 Envelope and MPER Antibody Structures in Lipid Assemblies. Cell Rep. 2020;31(4):107583. doi: 10.1016/j.celrep.2020.107583 ; PubMed Central PMCID: PMC7196886. 32348769 PMC7196886

[83] Kwon YD, Pancera M, Acharya P, Georgiev IS, Crooks ET, Gorman J, et al. Crystal structure, conformational fixation and entry-related interactions of mature ligand-free HIV-1 Env. Nat Struct Mol Biol. 2015;22(7):522–31. Epub 20150622. doi: 10.1038/nsmb.3051 ; PubMed Central PMCID: PMC4706170. 26098315 PMC4706170

[84] Castillo-Menendez LR, Nguyen HT, Sodroski J. Conformational Differences between Functional Human Immunodeficiency Virus Envelope Glycoprotein Trimers and Stabilized Soluble Trimers. J Virol. 2019;93(3). Epub 20190117. doi: 10.1128/JVI.01709-18 ; PubMed Central PMCID: PMC6340038. 30429345 PMC6340038

[85] Bjorndal A, Deng H, Jansson M, Fiore JR, Colognesi C, Karlsson A, et al. Coreceptor usage of primary human immunodeficiency virus type 1 isolates varies according to biological phenotype. J Virol. 1997;71(10):7478–87. doi: 10.1128/JVI.71.10.7478-7487.1997 PubMed Central PMCID: PMC192094. 9311827 PMC192094

[86] Purtscher M, Trkola A, Grassauer A, Schulz PM, Klima A, Dopper S, et al. Restricted antigenic variability of the epitope recognized by the neutralizing gp41 antibody 2F5. AIDS. 1996;10(6):587–93. doi: 10.1097/00002030-199606000-00003 . 8780812

[87] Stiegler G, Kunert R, Purtscher M, Wolbank S, Voglauer R, Steindl F, et al. A potent cross-clade neutralizing human monoclonal antibody against a novel epitope on gp41 of human immunodeficiency virus type 1. AIDS Res Hum Retroviruses. 2001;17(18):1757–65. doi: 10.1089/08892220152741450 . 11788027

[88] Huang J, Ofek G, Laub L, Louder MK, Doria-Rose NA, Longo NS, et al. Broad and potent neutralization of HIV-1 by a gp41-specific human antibody. Nature. 2012;491(7424):406–12. Epub 20120918. doi: 10.1038/nature11544 ; PubMed Central PMCID: PMC4854285. 23151583 PMC4854285

[89] Kwon YD, Georgiev IS, Ofek G, Zhang B, Asokan M, Bailer RT, et al. Optimization of the Solubility of HIV-1-Neutralizing Antibody 10E8 through Somatic Variation and Structure-Based Design. J Virol. 2016;90(13):5899–914. Epub 20160610. doi: 10.1128/JVI.03246-15 ; PubMed Central PMCID: PMC4907239. 27053554 PMC4907239

[90] Burton DR, Barbas CF 3rd, Persson MA, Koenig S, Chanock RM, Lerner RA. A large array of human monoclonal antibodies to type 1 human immunodeficiency virus from combinatorial libraries of asymptomatic seropositive individuals. Proc Natl Acad Sci U S A. 1991;88(22):10134–7. Epub 1991/11/15. doi: 10.1073/pnas.88.22.10134 PubMed Central PMCID: PMC52882. 1719545 PMC52882

[91] Wu X, Yang ZY, Li Y, Hogerkorp CM, Schief WR, Seaman MS, et al. Rational design of envelope identifies broadly neutralizing human monoclonal antibodies to HIV-1. Science. 2010;329(5993):856–61. Epub 20100708. doi: 10.1126/science.1187659 ; PubMed Central PMCID: PMC2965066. 20616233 PMC2965066

[92] Posner MR, Cavacini LA, Emes CL, Power J, Byrn R. Neutralization of HIV-1 by F105, a human monoclonal antibody to the CD4 binding site of gp120. J Acquir Immune Defic Syndr (1988). 1993;6(1):7–14. Epub 1993/01/01. . 8417177

[93] Thali M, Moore JP, Furman C, Charles M, Ho DD, Robinson J, et al. Characterization of conserved human immunodeficiency virus type 1 gp120 neutralization epitopes exposed upon gp120-CD4 binding. J Virol. 1993;67(7):3978–88. doi: 10.1128/JVI.67.7.3978-3988.1993 ; PubMed Central PMCID: PMC237765. 7685405 PMC237765

[94] Buchacher A, Predl R, Strutzenberger K, Steinfellner W, Trkola A, Purtscher M, et al. Generation of human monoclonal antibodies against HIV-1 proteins; electrofusion and Epstein-Barr virus transformation for peripheral blood lymphocyte immortalization. AIDS Res Hum Retroviruses. 1994;10(4):359–69. Epub 1994/04/01. doi: 10.1089/aid.1994.10.359 . 7520721

[95] Gorny MK, Conley AJ, Karwowska S, Buchbinder A, Xu JY, Emini EA, et al. Neutralization of diverse human immunodeficiency virus type 1 variants by an anti-V3 human monoclonal antibody. J Virol. 1992;66(12):7538–42. doi: 10.1128/JVI.66.12.7538-7542.1992 ; PubMed Central PMCID: PMC240465. 1433529 PMC240465

[96] Gorny MK, Xu JY, Karwowska S, Buchbinder A, Zolla-Pazner S. Repertoire of neutralizing human monoclonal antibodies specific for the V3 domain of HIV-1 gp120. J Immunol. 1993;150(2):635–43. . 7678279

[97] Huang J, Kang BH, Pancera M, Lee JH, Tong T, Feng Y, et al. Broad and potent HIV-1 neutralization by a human antibody that binds the gp41-gp120 interface. Nature. 2014;515(7525):138–42. Epub 20140903. doi: 10.1038/nature13601 ; PubMed Central PMCID: PMC4224615. 25186731 PMC4224615

[98] Walker LM, Phogat SK, Chan-Hui PY, Wagner D, Phung P, Goss JL, et al. Broad and potent neutralizing antibodies from an African donor reveal a new HIV-1 vaccine target. Science. 2009;326(5950):285–9. Epub 20090903. doi: 10.1126/science.1178746 ; PubMed Central PMCID: PMC3335270. 19729618 PMC3335270

[99] Walker LM, Huber M, Doores KJ, Falkowska E, Pejchal R, Julien JP, et al. Broad neutralization coverage of HIV by multiple highly potent antibodies. Nature. 2011;477(7365):466–70. doi: 10.1038/nature10373 ; PubMed Central PMCID: PMC3393110. 21849977 PMC3393110

[100] Shingai M, Nishimura Y, Klein F, Mouquet H, Donau OK, Plishka R, et al. Antibody-mediated immunotherapy of macaques chronically infected with SHIV suppresses viraemia. Nature. 2013;503(7475):277–80. Epub 20131030. doi: 10.1038/nature12746 ; PubMed Central PMCID: PMC4133787. 24172896 PMC4133787

[101] Bell CH, Pantophlet R, Schiefner A, Cavacini LA, Stanfield RL, Burton DR, et al. Structure of antibody F425-B4e8 in complex with a V3 peptide reveals a new binding mode for HIV-1 neutralization. J Mol Biol. 2008;375(4):969–78. Epub 20071113. doi: 10.1016/j.jmb.2007.11.013 ; PubMed Central PMCID: PMC2289799. 18068724 PMC2289799

[102] Baba M, Nishimura O, Kanzaki N, Okamoto M, Sawada H, Iizawa Y, et al. A small-molecule, nonpeptide CCR5 antagonist with highly potent and selective anti-HIV-1 activity. Proc Natl Acad Sci U S A. 1999;96(10):5698–703. doi: 10.1073/pnas.96.10.5698 ; PubMed Central PMCID: PMC21923. 10318947 PMC21923

[103] Stamatatos L, Wiskerchen M, Cheng-Mayer C. Effect of major deletions in the V1 and V2 loops of a macrophage-tropic HIV type 1 isolate on viral envelope structure, cell entry, and replication. AIDS Res Hum Retroviruses. 1998;14(13):1129–39. Epub 1998/09/16. doi: 10.1089/aid.1998.14.1129 . 9737584

[104] Wu X, Parast AB, Richardson BA, Nduati R, John-Stewart G, Mbori-Ngacha D, et al. Neutralization escape variants of human immunodeficiency virus type 1 are transmitted from mother to infant. J Virol. 2006;80(2):835–44. Epub 2005/12/28. doi: 10.1128/JVI.80.2.835-844.2006 ; PubMed Central PMCID: PMC1346878. 16378985 PMC1346878

[105] Adachi A, Gendelman HE, Koenig S, Folks T, Willey R, Rabson A, et al. Production of acquired immunodeficiency syndrome-associated retrovirus in human and nonhuman cells transfected with an infectious molecular clone. J Virol. 1986;59(2):284–91. Epub 1986/08/01. doi: 10.1128/JVI.59.2.284-291.1986 ; PubMed Central PMCID: PMC253077. 3016298 PMC253077

[106] Lewis N, Williams J, Rekosh D, Hammarskjold ML. Identification of a cis-acting element in human immunodeficiency virus type 2 (HIV-2) that is responsive to the HIV-1 rev and human T-cell leukemia virus types I and II rex proteins. J Virol. 1990;64(4):1690–7. Epub 1990/04/01. doi: 10.1128/JVI.64.4.1690-1697.1990 ; PubMed Central PMCID: PMC249306. 2157051 PMC249306

[107] Chen BK, Saksela K, Andino R, Baltimore D. Distinct modes of human immunodeficiency virus type 1 proviral latency revealed by superinfection of nonproductively infected cell lines with recombinant luciferase-encoding viruses. J Virol. 1994;68(2):654–60. doi: 10.1128/JVI.68.2.654-660.1994 ; PubMed Central PMCID: PMC236499. 7507183 PMC236499

[108] Edelhoch H. Spectroscopic determination of tryptophan and tyrosine in proteins. Biochemistry. 1967;6(7):1948–54. doi: 10.1021/bi00859a010 . 6049437

[109] Lee S, Lee DK. What is the proper way to apply the multiple comparison test? Korean J Anesthesiol. 2018;71(5):353–60. Epub 20180828. doi: 10.4097/kja.d.18.00242 ; PubMed Central PMCID: PMC6193594. 30157585 PMC6193594

