# Supplementary material for: Regulation of epitope exposure in the gp41 membrane-proximal external region through interactions at the apex of HIV-1 Env
Source: PLoS Pathog. 2022 May 18;18(5):e1010531. doi: 10.1371/journal.ppat.1010531 (PMC9154124; doi:10.1371/journal.ppat.1010531)
Supplement: S1 Table — Numbers reflect the geometric means obtained from viruses with both detected and undetected IC50 values. Orange designates geometric means calculated from groups in which less than 85% of the IC50 values were measurable. Data were procured from the Los Alamos National Laboratory HIV Immunology database (January 2022). For comparison, the mean IC50 values for H4, H5 and H4 R306S Envs found in this study are shown. Red numbers indicate that 50% neutralization was not achieved at the highest concentrations tested. (DOCX) [file ppat.1010531.s001.docx]

**S1 Table. IC50 values (μg/ml) for different NAbs grouped by tier designation.** Numbers reflect the geometric means obtained from viruses with both detected and undetected IC50 values. Orange designates geometric means calculated from groups in which less than 85% of the IC50 values were measurable. Data were procured from the Los Alamos National Laboratory HIV Immunology database (January 2022). For comparison, the mean IC50 values for H4, H5 and H4 R306S Envs found in this study are shown. Red numbers indicate that 50% neutralization was not achieved at the highest concentrations tested.

| Antibody | **2F5** | **10E8** | **10E8v4** | **4E10** | **35O22** | **2G12** | **PG9** | **PG16** | **PGT145** |
| --- | --- | --- | --- | --- | --- | --- | --- | --- | --- |
| Epitope | **MPER (662-668, linear)** | **MPER (671-683, linear)** | **MPER (671-683, linear)** | **MPER (671-677, linear)** | **gp120-gp41 interface (4^o^)** | **Lateral apical glycans (3^o^)** | **V1/V2 loop (4^o^)** | **V1/V2 loop (4^o^)** | **Trimer apex (4^o^)** |
| Reference | [1] | [1] | [1] | [1] | [2] | [2] | [2] | [3] | [2] |
| **1A** | 0.747 | 0.012 | 0.018 | 0.114 | 0.775 | 6.364 | 3.384 | 1.606 | 1.666 |
| **1B** | 21.507 | 0.234 | 0.073 | 1.853 | 0.018 | 36.306 | 0.211 | 0.225 | 0.501 |
| **2** | 25.325 | 0.499 | 0.445 | 3.179 | 5.007 | 55.772 | 0.747 | 0.558 | 0.797 |
| **2B** | 4.355 | 0.215 | 0.329 | 2.323 | 3.247 | 11.855 | 2.800 | 3.484 | 0.511 |
| **3** | 83.070 | 0.933 | 2.653 | 12.284 | 13.317 | 84.381 | 2.290 | 1.912 | 3.258 |
| **H4** | 0.293 | 0.0529 | 0.0588 | 0.0579 | 2.68 | 0.79 | 22 | 40 | 100 |
| **H5** | 6.46 | 1.23 | 0.751 | 1.19 | 31.4 | 0.681 | 6.3 | 40 | 100 |
| **H4 R306S** | 3.05 | 0.833 | 1.34 | nd* | 20.9 | 0.464 | 50 | 40 | 100 |

| Antibody | **b12** | **VRC01** | **F105** | **sCD4^#^** | **447-52D** | **10-1074^†^** | **F425-B4e8^†^** | **257- D IV^†^** | **17b** |
| --- | --- | --- | --- | --- | --- | --- | --- | --- | --- |
| Epitope | **CD4 Binding Site (3^o^)** | **CD4 Binding Site (3^o^)** | **CD4 Binding Site (3^o^)** | **CD4 Binding Site (3^o^)** | **V3-Loop Crown (312-315, GPGR, linear)** | **N332 glycan + V3 loop base (325-328, GDIR, 3^o^)** | **V3-Loop Crown (309-315, IGPGR)** | **V3 Loop (305-309, KRIHI, linear)** | **gp120 Bridging Sheet (3^o^)** |
| Reference | [2] | [2] | [4] | [5] | [1] | [6] | [7] | [1] | [5] |
| **1A** | 0.453 | 0.168 | 41.338 | 0.133 | 0.242 | 0.079 | na^ | na | 1.358 |
| **1B** | 15.825 | 0.305 | 8.223 | 0.451 | 1.051 | 0.010 | na | na | 18.447 |
| **2** | 31.033 | 0.963 | 92.485 | 7.489 | 73.154 | 0.639 | na | na | 94.242 |
| **2B** | 5.763 | 0.522 | 83.918 | 7.165 | 46.520 | 0.081 | na | na | 96.833 |
| **3** | 63.538 | 4.325 | 100.000 | 7.027 | 100.000 | 3.092 | na | na | 100.000 |
| **H4** | 0.0256 | 0.163 | 0.687 | 0.036 | 0.807 | 4 | 40 | 15.4 | 2.97 |
| **H5** | 1.33 | 1.48 | 10 | 1.8 | 40 | 0.0727 | 40 | 40 | 1000 |
| **H4 R306S** | 0.0718 | 0.346 | 2.7 | 0.18 | 40 | 4 | 40 | 29.2 | 22.4 |

*nd designates “not determined.”

^na designates “not available”

^#^Values are based on the molecular weight of 2-domain sCD4 used in this study

**^†^**V3-loop residues in the epitope sequences significantly differ between Env_HXB2_ and Env_SF162_

References

1. HIV Molecular Immunology Database: Los Alamos National Laboratories; 2021 [January 2022].

2. Chuang GY, Zhou J, Acharya P, Rawi R, Shen CH, Sheng Z, et al. Structural Survey of Broadly Neutralizing Antibodies Targeting the HIV-1 Env Trimer Delineates Epitope Categories and Characteristics of Recognition. Structure. 2019;27(1):196-206 e6. Epub 20181121. doi: 10.1016/j.str.2018.10.007. PubMed PMID: 30471922; PubMed Central PMCID: PMC6664815.

3. Pancera M, Shahzad-Ul-Hussan S, Doria-Rose NA, McLellan JS, Bailer RT, Dai K, et al. Structural basis for diverse N-glycan recognition by HIV-1-neutralizing V1-V2-directed antibody PG16. Nat Struct Mol Biol. 2013;20(7):804-13. Epub 20130526. doi: 10.1038/nsmb.2600. PubMed PMID: 23708607; PubMed Central PMCID: PMC4046252.

4. Pancera M, Changela A, Kwong PD. How HIV-1 entry mechanism and broadly neutralizing antibodies guide structure-based vaccine design. Curr Opin HIV AIDS. 2017;12(3):229-40. Epub 2017/04/20. doi: 10.1097/COH.0000000000000360. PubMed PMID: 28422787; PubMed Central PMCID: PMC5557343.

5. Kwong PD, Wyatt R, Robinson J, Sweet RW, Sodroski J, Hendrickson WA. Structure of an HIV gp120 envelope glycoprotein in complex with the CD4 receptor and a neutralizing human antibody. Nature. 1998;393(6686):648-59. doi: 10.1038/31405. PubMed PMID: 9641677; PubMed Central PMCID: PMC5629912.

6. Gristick HB, von Boehmer L, West AP, Jr., Schamber M, Gazumyan A, Golijanin J, et al. Natively glycosylated HIV-1 Env structure reveals new mode for antibody recognition of the CD4-binding site. Nat Struct Mol Biol. 2016;23(10):906-15. Epub 20160912. doi: 10.1038/nsmb.3291. PubMed PMID: 27617431; PubMed Central PMCID: PMC5127623.

7. Bell CH, Pantophlet R, Schiefner A, Cavacini LA, Stanfield RL, Burton DR, et al. Structure of antibody F425-B4e8 in complex with a V3 peptide reveals a new binding mode for HIV-1 neutralization. J Mol Biol. 2008;375(4):969-78. Epub 20071113. doi: 10.1016/j.jmb.2007.11.013. PubMed PMID: 18068724; PubMed Central PMCID: PMC2289799.
