# Supplementary material for: Regulation of epitope exposure in the gp41 membrane-proximal external region through interactions at the apex of HIV-1 Env
Source: PLoS Pathog. 2022 May 18;18(5):e1010531. doi: 10.1371/journal.ppat.1010531 (PMC9154124; doi:10.1371/journal.ppat.1010531)
Supplement: S2 Table — Numbers reflect the mean and SEM from three or more independent experiments. The units are ng/ml for all NAbs and nM for sCD4. Red numbers indicate that 50% neutralization was not achieved at the highest concentrations tested. Blank spaces indicate that the antibody or sCD4 was not tested against that Env. Note that B4 R306S Env was nonfunctional. (DOCX) [file ppat.1010531.s002.docx]

**S2 Table. IC50 values for different NAbs and sCD4 against Envs with V3-loop swaps and residue 306 substitutions.** Numbers reflect the mean and SEM from three or more independent experiments. The units are ng/ml for all NAbs and nM for sCD4. Red numbers indicate that 50% neutralization was not achieved at the highest concentrations tested. Blank spaces indicate that the antibody or sCD4 was not tested against that Env. Note that B4 R306S Env was nonfunctional.

|  |  |  |  |  |  |  |  |  |  |  |  |  |  |  |  |  |  |  |
| --- | --- | --- | --- | --- | --- | --- | --- | --- | --- | --- | --- | --- | --- | --- | --- | --- | --- | --- |
|  | **2F5** | **SEM** | **10E8** | **SEM** | **10E8v4** | **SEM** | **4E10** | **SEM** | **35O22** | **SEM** | **2G12** | **SEM** | **PG9** | **SEM** | **PG16** | **SEM** | **PGT145** | **SEM** |
| **H4** | 293 | 34.8 | 52.9 | 3.17 | 58.8 | 6.2 | 57.9 | 6.8 | 2680 | 577 | 790 | 88.7 | 22000 | 6620 | 40000 |  | 1E+05 |  |
| **H4 R306S** | 3050 | 382 | 833 | 73.3 | 1340 | 128 |  |  | 20900 | 2420 | 464 | 53.0 | 50000 |  | 40000 |  | 1E+05 |  |
| **H5** | 6460 | 1730 | 1227 | 175 | 751 | 89.3 | 1190 | 146 | 31400 | 397 | 681 | 144 | 6300 | 2350 | 40000 |  | 1E+05 |  |
| **H5 S306R** |  |  | 1197 | 406 |  |  |  |  |  |  | 532 | 94.5 |  |  |  |  |  |  |
| **N4 S306R** |  |  | 95.1 | 10.7 | 129 | 12.8 |  |  |  |  | 701 | 108 |  |  |  |  |  |  |
| **N4** | 2300 | 500 | 351 | 84.2 | 941 | 74.5 |  |  |  |  | 202 | 27.2 |  |  |  |  |  |  |
| **N5** | 1100 | 400 | 541 | 113 |  |  |  |  |  |  | 467 | 112 |  |  |  |  |  |  |
| **N5 S306R** |  |  | 456 | 61.5 |  |  |  |  |  |  | 296 | 76.2 |  |  |  |  |  |  |
| **B4** |  |  | 62.6 | 4.61 |  |  |  |  |  |  | 1100 | 82.3 |  |  |  |  |  |  |
| **B4 R306S** |  |  |  |  |  |  |  |  |  |  |  |  |  |  |  |  |  |  |
| **B5** |  |  | 1237 | 84.3 |  |  |  |  |  |  | 20000 |  |  |  |  |  |  |  |
| **B5 S306R** |  |  | 1146 | 132 |  |  |  |  |  |  | 20000 |  |  |  |  |  |  |  |
| **J4** |  |  | 213 | 21.3 | 188 | 23.6 |  |  | 737 | 200 | 118 | 6.91 | 40000 |  | 40000 |  | 40000 |  |
| **J4 R306S** |  |  | 243 | 11.3 |  |  |  |  |  |  | 88 | 24.9 |  |  |  |  |  |  |
| **J5** |  |  | 758 | 74.1 | 806 | 63.4 |  |  | 131 | 60 | 283 | 55.8 | 20000 |  | 40000 |  | 20800 | 2120 |
| **J5 S306R** |  |  | 622 | 97.8 |  |  |  |  |  |  | 140 | 8.29 |  |  |  |  |  |  |
| **S4** |  |  | 245 | 12.5 |  |  |  |  | 40000 |  | 552 | 197 | 40000 |  | 40000 |  | 40000 |  |
| **S4 R306S** |  |  | 229 | 57.2 |  |  |  |  |  |  | 569 | 62.5 |  |  |  |  |  |  |
| **S5** |  |  | 1341 | 28.4 |  |  |  |  | 40000 |  | 456 | 159 | 40000 |  | 40000 |  | 40000 |  |
| **S5 S306R** |  |  | 1174 | 213 |  |  |  |  |  |  | 492 | 207 |  |  |  |  |  |  |

|  |  |  |  |  |  |  |  |  |  |  |  |  |  |  |  |  |  |  |
| --- | --- | --- | --- | --- | --- | --- | --- | --- | --- | --- | --- | --- | --- | --- | --- | --- | --- | --- |
|  | **b12** | **SEM** | **VRC01** | **SEM** | **F105** | **SEM** | **sCD4** | **SEM** | **447-52D** | **SEM** | **10-1074** | **SEM** | **F425-B4e8** | **SEM** | **257-D IV** | **SEM** | **17b** | **SEM** |
| **H4** | 25.6 | 5.43 | 163 | 17.4 | 687 | 150 | 1.40 | 0.21 | 807 | 181 | 4000 |  | 40000 |  | 15400 | 1790 | 2970 | 1410 |
| **H4 R306S** | 71.8 | 23.6 | 346 | 23.6 | 2700 | 800 | 7.05 | 1.33 | 40000 |  | 4000 |  | 40000 |  | 29200 | 5520 | 22400 | 6410 |
| **H5** | 1331 | 388 | 1483 | 309 | 10000 |  | 70.8 | 8.76 | 40000 |  | 72.7 | 14.4 | 40000 |  | 40000 |  | 1E+06 |  |
| **H5 S306R** | 1345 | 117 | 755 | 72.5 | 10000 |  |  |  |  |  |  |  |  |  |  |  | 1E+06 |  |
| **N4 S306R** | 13.8 | 3.19 | 201 | 33.8 | 745 | 130 |  |  |  |  |  |  |  |  |  |  | 133 | 8.82 |
| **N4** | 180 | 79.1 | 179 | 44.3 | 656 | 247 |  |  |  |  |  |  |  |  |  |  | 107 | 13.3 |
| **N5** | 1772 | 359 | 1027 | 137 | 10000 |  |  |  |  |  |  |  |  |  |  |  | 1E+06 |  |
| **N5 S306R** | 911 | 32.3 | 419 | 44.2 | 10000 |  |  |  |  |  |  |  |  |  |  |  | 1E+06 |  |
| **B4** | 48.4 | 10.1 | 88.1 | 4.03 | 915 | 247 |  |  |  |  |  |  |  |  |  |  | 567 | 78.4 |
| **B4 R306S** |  |  |  |  |  |  |  |  |  |  |  |  |  |  |  |  |  |  |
| **B5** | 20000 |  | 151 | 17.0 | 10000 |  |  |  |  |  |  |  |  |  |  |  | 1E+06 |  |
| **B5 S306R** | 20000 |  | 89.4 | 18.5 | 10000 |  |  |  |  |  |  |  |  |  |  |  | 1E+06 |  |
| **J4** | 12.0 | 3.37 | 23 | 4.15 | 557 | 53.2 |  |  | 46.3 | 3.71 | 4000 |  | 40000 |  | 25600 | 5450 | 2.E+05 | 36800 |
| **J4 R306S** | 10.2 | 1.63 | 28.3 | 8.14 | 1460 | 150 |  |  |  |  |  |  |  |  |  |  | 6.E+05 | 1.E+05 |
| **J5** | 61.3 | 5.63 | 45.0 | 5.03 | 10000 |  | 201 | 24.4 | 40000 |  | 71 | 6.56 | 20000 |  | 13900 | 2470 | 1E+06 |  |
| **J5 S306R** | 29.2 | 10.3 | 28.1 | 3.65 | 10000 |  |  |  |  |  |  |  |  |  |  |  | 1E+06 |  |
| **S4** | 15 | 1.56 | 426 | 76.9 | 232 | 83.1 |  |  | 27 | 2.08 | 2000 |  | 3250 | 1610 | 26300 | 9500 | 1060 | 122 |
| **S4 R306S** | 15.9 | 4.70 | 579 | 176 | 323 | 243 |  |  |  |  |  |  |  |  |  |  | 2200 | 720 |
| **S5** | 287 | 55.9 | 610 | 107 | 10000 |  |  |  | 18700 | 7030 | 23.7 | 3.67 | 1890 | 592 | 9490 | 2260 | 1E+06 |  |
| **S5 S306R** | 270 | 74.1 | 235 | 39.1 | 10000 |  |  |  |  |  |  |  |  |  |  |  | 6E+05 | 1E+05 |

**Table S2 (cont)**
