# Supplementary figures and images for: Regulation of epitope exposure in the gp41 membrane-proximal external region through interactions at the apex of HIV-1 Env
Source: PLoS Pathog. 2022 May 18;18(5):e1010531. doi: 10.1371/journal.ppat.1010531 (PMC9154124; doi:10.1371/journal.ppat.1010531)

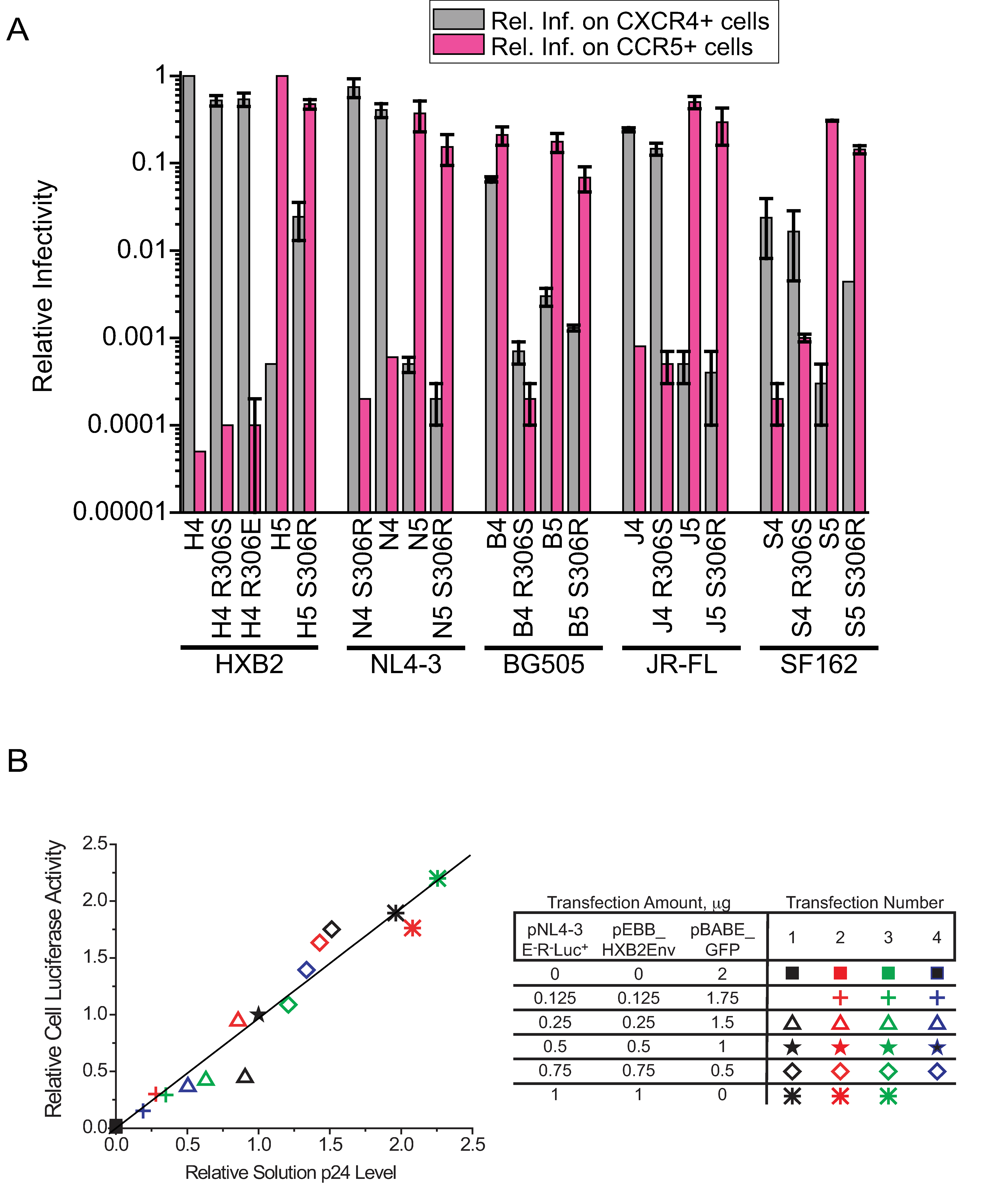

Supplement: S1 Fig — (A) For each virus, infectivity was measured on both U87.CD4.CXCR4 cells (grey bars) and U87.CD4.CCR5 (magenta bars) and normalized to viral content estimated by luciferase expression from proviral DNA in virus-producing cells (see B). Normalized infectivity is reported relative to the values for H4 Env on CXCR4+ cells and H5 Env on CCR5+ cells. Data represent the mean±range-of-mean from two independent experiments. (B) Relationship between luciferase expression from virus producing cells and HIV-1 p24 levels found in culture supernatants. For each experiment, cells were transfected (Lipofectamine Plus, Life Sciences) with differing amounts of pseudotyping vector pNL4-3 E-R-Luc+, Env-expressing vector pEBB_HXB2Env and GFP-expressing vector pBABE_GFP as indicated in the legend. After a 36 hour incubation, supernatants were collected and assayed for viral p24 content by ELISA (see reference [63] of text) while cells were lysed and assessed for luciferase activity. For each experiment, the measured solution p24 levels and luciferase activities were normalized to their respective values obtained from the 0.5 μg pNL4-3 E-R-Luc+ transfection. Data from four separate experiments have been fit to a linear regression fixed to go through the origin; the slope was 0.96 and the R-value was 0.93. (TIF) [file ppat.1010531.s003.tif]

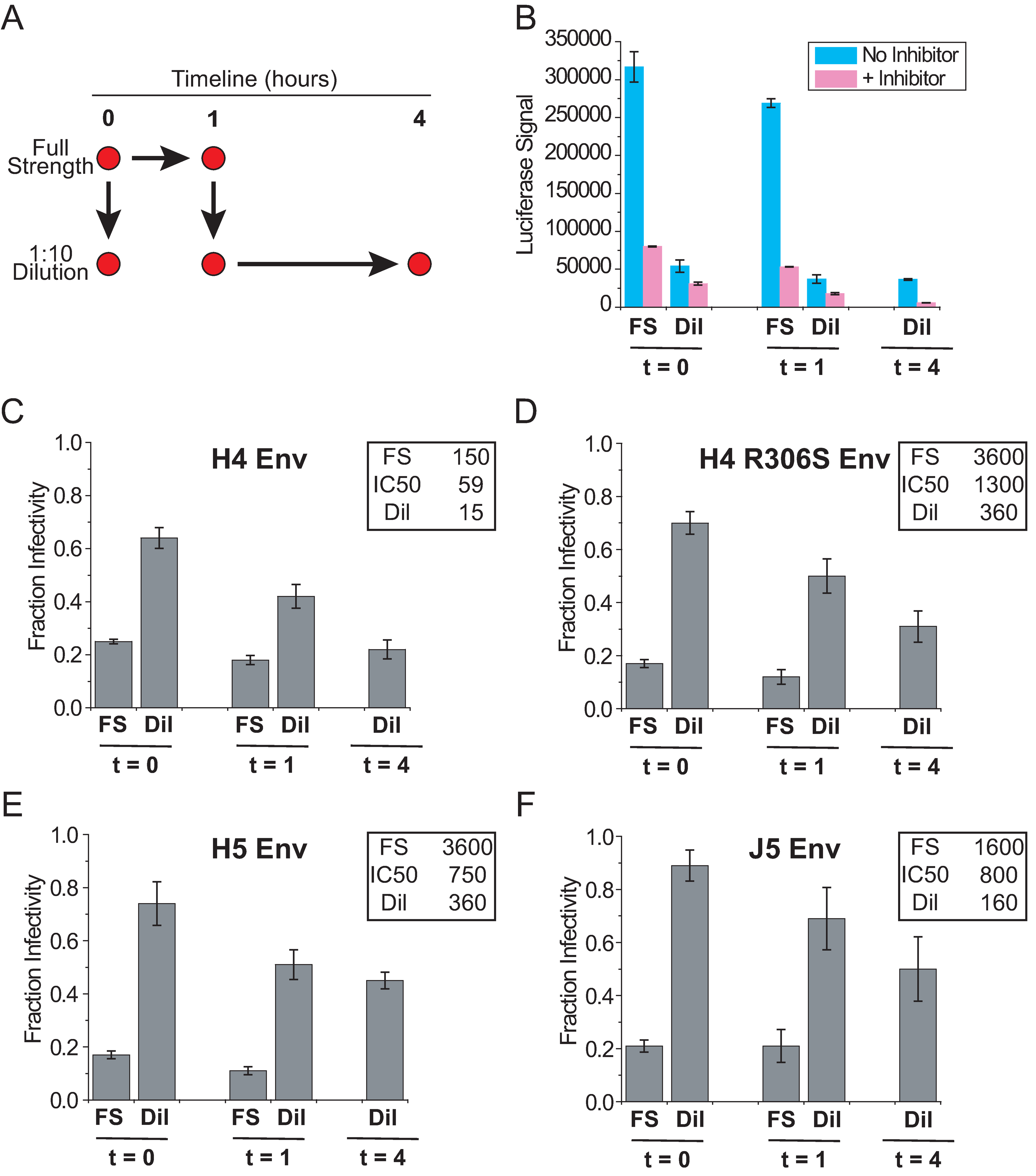

Supplement: S2 Fig — (A) Experimental timeline of the antibody dilution experiment. HIV-1 was suspended in media with and without NAb 10E8v4 at 2-4x IC50 concentration to form the full-strength samples. A portion of these samples (± antibody) was diluted 10-fold either immediately or after a 1-hour incubation at 37°C. The sample diluted at 1 hour was further incubated at 37°C for 3 hours to allow for antibody unbinding and recovery from neutralization. Culture media over U87 target cells was completely replaced with full strength or diluted samples at the 0-, 1- and 4- hour time points (red circles) to asses viral infectivity in the presence or absence of antibody. (B) Representative data from a single experiment using HIV-1 pseudotyped with H4 Env. Infectivity was measured by luciferase reporter expression for full strength (FS) or diluted (Dil) samples at the indicated time points. Bars and error bars represent the mean ± SEM of three experimental replicates for each sample. (C-F) NAb 10E8v4 neutralization of HIV-1 pseudotyped with H4 (C), H4 R306S (D), H5 (E) and J5 (F) Envs. Fraction infectivity was calculated as the ratio of the luciferase signals recorded in the presence and absence of NAb 10E8v4 (i.e., adjacent magenta and cyan bars in B) under each condition (FS or Dil) at the indicated time point. Antibody concentrations (ng/mL) in the FS and Dil conditions, as well as the IC50 value, are listed in the adjacent box. Data represent the mean ± SEM from four to five independent experiments. The lower fraction infectivity at the 1-hour timepoint compared to the 0-hour timepoint (more easily seen in the Dil samples) indicated that antibody binding to the prefusogenic state occurred during the 0-to-1 hour incubation period under FS conditions. Neutralization was enhanced by approximately the same amount for all Envs tested despite the fact that NAb 10E8 concentrations in H4 R306S, H5 and J5 Env viral samples were approximately 10- to 20-fold higher than that in wild type H4 Env sampl [file ppat.1010531.s004.tif]

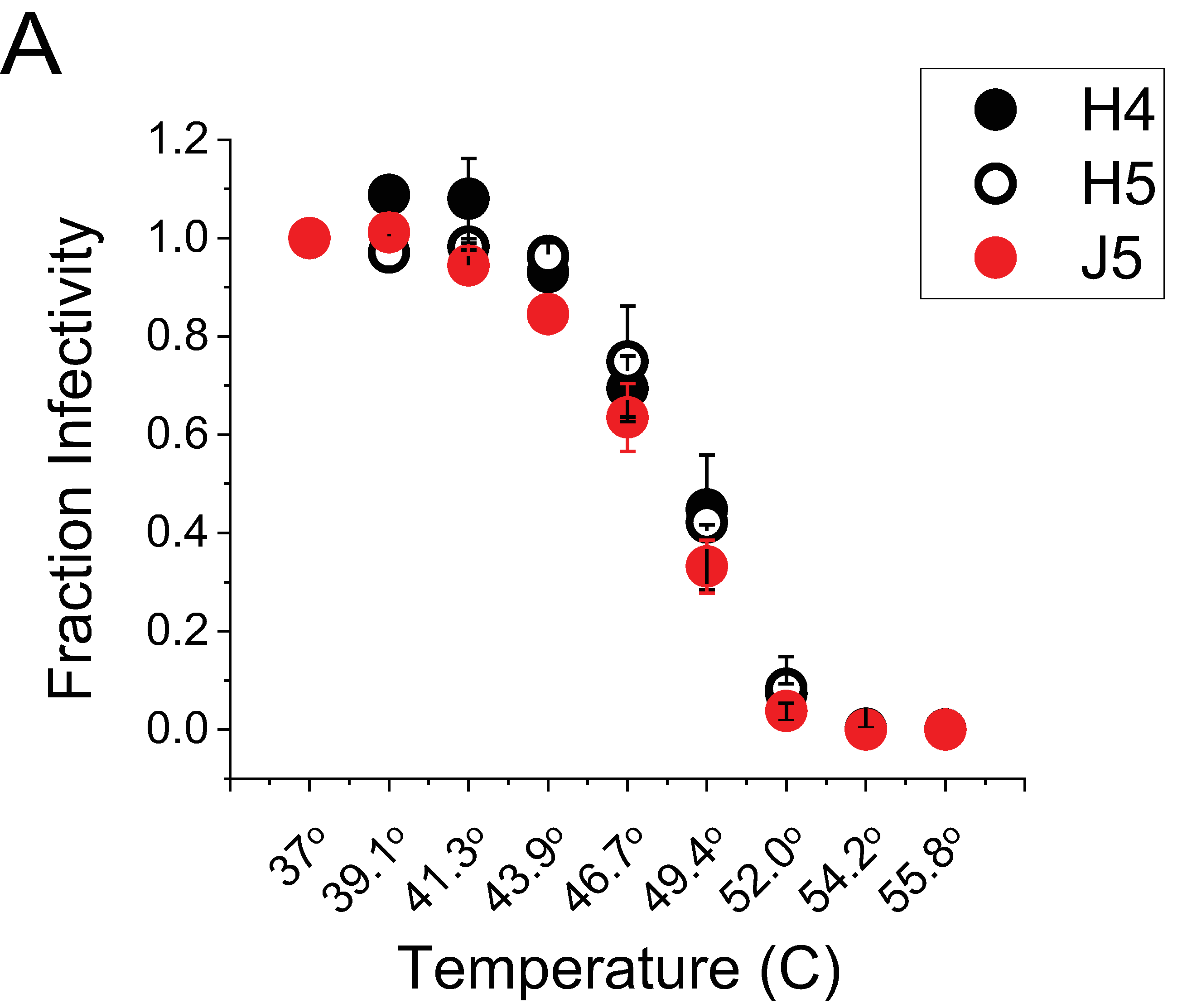

Supplement: S3 Fig — Viral aliquots were incubated at various temperatures for 45 minutes prior to addition to U87 cell cultures at 37°C. Infectivity levels were normalized to the level measured following a 37°C incubation. Data represent the mean±range-of-mean from two independent experiments. (TIF) [file ppat.1010531.s005.tif]

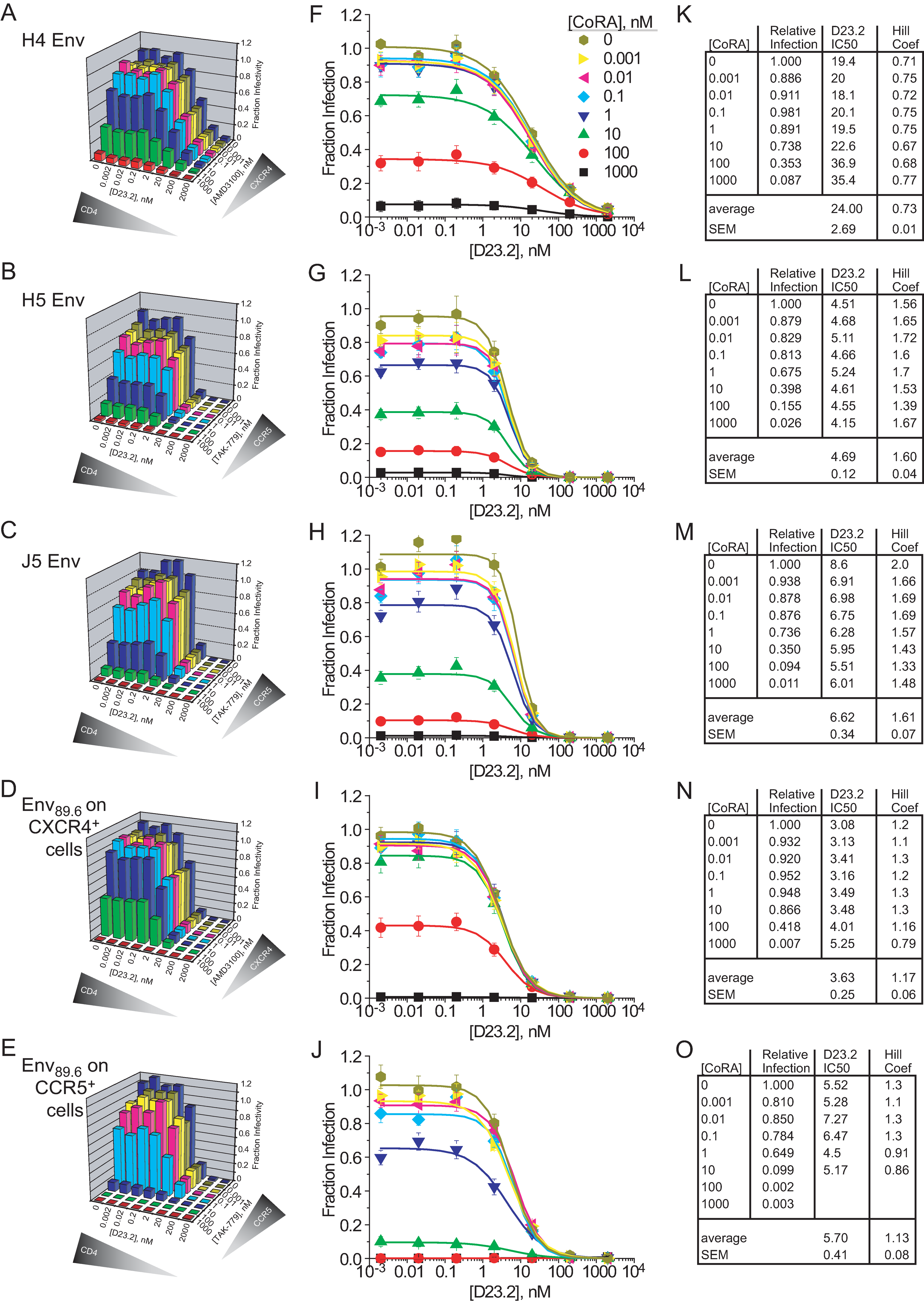

Supplement: S4 Fig — (A-E) Infectivity levels of pseudotyped HIV-1 were measured in the presence of CD4 antagonist (D23.2) or CoRA (AMD3100 or TAK-779) alone or in combination. U87.CD4.CXCR4 target cells were used for H4 Env (A) and Env89.6 (D), while U87.CD4.CCR5 target cells were used for H5 Env (B), J5 Env (C) and Env89.6 (E). Data were normalized to the level obtained in the absence of both inhibitors. (F-J) The same data in A-E except plotted as D23.2 titrations for each CoRA concentration. Data represent the mean±SEM from seven independent experiments and have been fit to the Hill Equation: FractionInfection=I01+([D32.2]IC50)nH where I0 represents the normalized infection level in the presence of CoRA but the absence of D23.2 and nH is the Hill coefficient. Data were reliably fit for relative infection levels greater than 0.005. (K-O) Relative infection level (Io), IC50 and Hill coefficient (nH) values extracted from the data fits in F-J. (TIF) [file ppat.1010531.s006.tif]

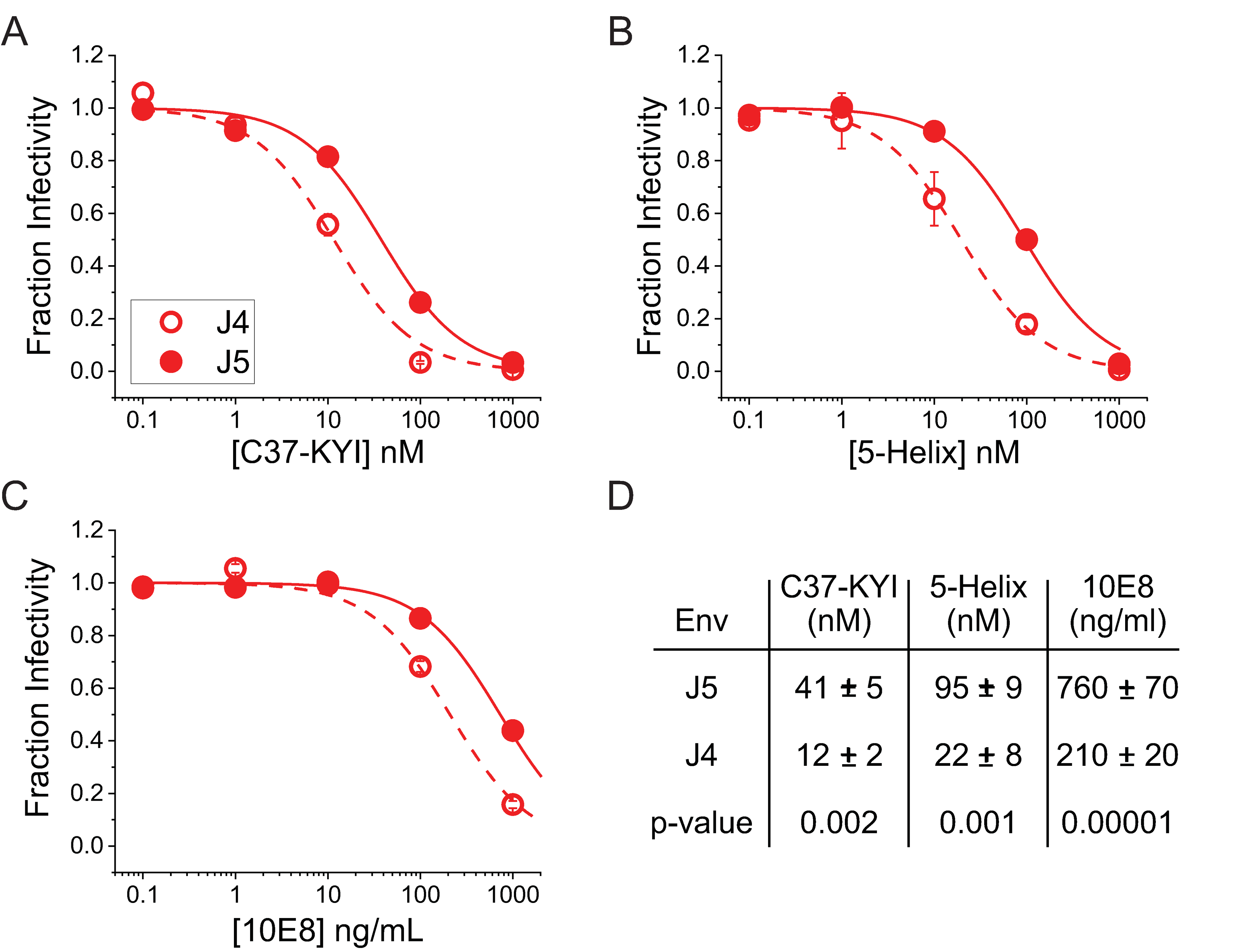

Supplement: S5 Fig — (A-C) Inhibitory titrations of C37-KYI (A), 5-Helix (B) and 10E8 (C) against HIV-1 pseudotyped with J4 (open) and J5 (closed) Envs. (D) Table of IC50 values. All data reflect the mean±SEM of four or more independent experiments. For all three inhibitors, J4 Env had significantly lower IC50 values than J5 Env (two-sample t-test with equal variance not assumed, p-value in table). (TIF) [file ppat.1010531.s007.tif]

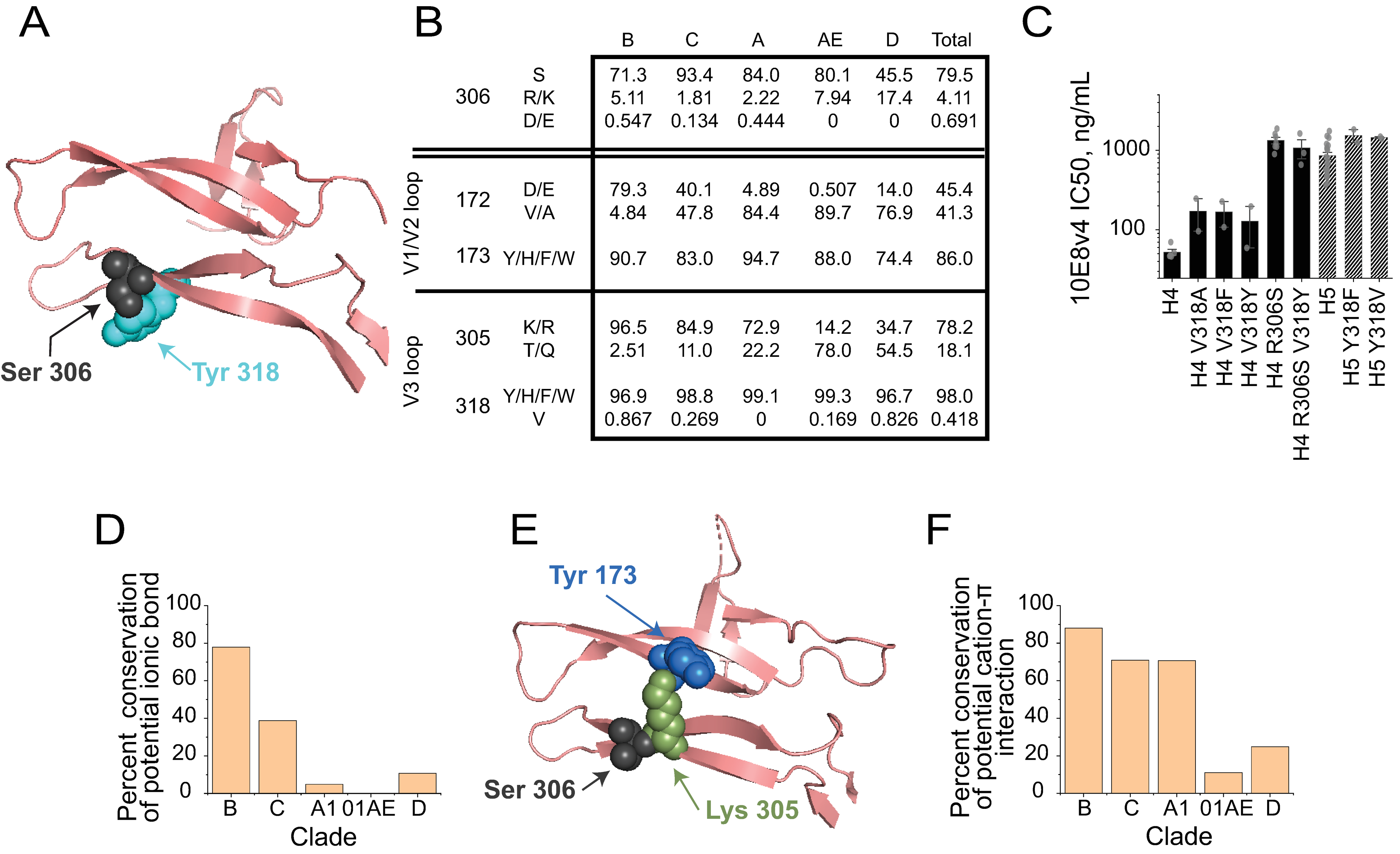

Supplement: S6 Fig — (A) Ribbon diagram of the V1/V2 and V3 loops in EnvJRFL SOSIP.664 (PDB 5FYK, reference [79] of main text) highlighting residues at position 306 (Ser-black) and 318 (Tyr-cyan). The same amino acids are found at positions 306 and 318 in H5 Env; in H4 Env, the amino acids are Arg306 and Val318. (B) Table of sequence conservation at V1/V2-loop residues 172 and 173 and V3-loop residues 305, 306 and 318 grouped by HIV-1 clade. Numbers represent summed frequencies of the indicated amino acids. (C) NAb 10E8v4 sensitivity of H4, H4 R306S and H5 Envs with the indicated substitutions at residue 318. Data from the Env variants with mutations at residue 318 represent the mean±range-of-mean from two independent experiments. Data from other variants represent mean±SEM of more than three independent experiments. (D) Frequency of a potential ionic interaction between V1/V2-loop residue 172 and V3-loop residue 305 grouped by clade. (E) Ribbon diagram of the V1/V2 and V3 loops in EnvBG505 SOSIP.664 structure (PDB 4ZMJ, reference [83] of main text) highlighting residues at position 173 (Tyr-blue), 305 (Lys-green) and 306 (Ser-black). (F) Frequency of a potential cation-pi interaction between V1/V2-loop residue 173 and V3-loop residue 305 grouped by clade. Sequence data obtained from the Los Alamos National Laboratory HIV Sequence database (October 2021). (TIF) [file ppat.1010531.s008.tif]
